# Bromodomain Protein Brd4 Plays a Key Role in Merkel Cell Polyomavirus DNA Replication

**DOI:** 10.1371/journal.ppat.1003021

**Published:** 2012-11-08

**Authors:** Xin Wang, Jing Li, Rachel M. Schowalter, Jing Jiao, Christopher B. Buck, Jianxin You

**Affiliations:** 1 Department of Microbiology, University of Pennsylvania Perelman School of Medicine, Philadelphia, Pennsylvania, United States of America; 2 Tumor Virus Molecular Biology Section, Laboratory of Cellular Oncology, National Cancer Institute, Bethesda, Maryland, United States of America; Fred Hutchinson Cancer Research Center, United States of America

## Abstract

Merkel cell polyomavirus (MCV or MCPyV) is the first human polyomavirus to be definitively linked to cancer. The mechanisms of MCV-induced oncogenesis and much of MCV biology are largely unexplored. In this study, we demonstrate that bromodomain protein 4 (Brd4) interacts with MCV large T antigen (LT) and plays a critical role in viral DNA replication. Brd4 knockdown inhibits MCV replication, which can be rescued by recombinant Brd4. Brd4 colocalizes with the MCV LT/replication origin complex in the nucleus and recruits replication factor C (RFC) to the viral replication sites. A dominant negative inhibitor of the Brd4-MCV LT interaction can dissociate Brd4 and RFC from the viral replication complex and abrogate MCV replication. Furthermore, obstructing the physiologic interaction between Brd4 and host chromatin with the chemical compound JQ1(+) leads to enhanced MCV DNA replication, demonstrating that the role of Brd4 in MCV replication is distinct from its role in chromatin-associated transcriptional regulation. Our findings demonstrate mechanistic details of the MCV replication machinery; providing novel insight to elucidate the life cycle of this newly discovered oncogenic DNA virus.

## Author Summary

MCV is a novel human polyomavirus that has recently been discovered in Merkel cell carcinoma (MCC), a rare but highly aggressive skin cancer. Several independent studies have confirmed that MCV is present in ∼80% of MCC tumors. However, very little is known about how the interaction between MCV and its human hosts contributes to the virus-induced cancers. Many aspects of the infectious life cycle of MCV are largely unexplored. In this study, we demonstrate that the MCV-encoded large T antigen can bind to host protein Brd4, which in turn serves as a scaffold that functionally recruits cellular DNA replication factors for replication of MCV viral DNA in host cells. This study is the first report to demonstrate mechanistic details of MCV's recruitment of the host cell DNA replication machinery; providing novel insight to elucidate the life cycle of this newly discovered oncogenic DNA virus. Importantly, our work demonstrates that blocking the Brd4 and MCV LT interaction can prevent MCV from replicating in host cells. This study identifies the Brd4-MCV LT interaction as an important target for potential development of effective therapeutic strategies to treat MCV infection.

## Introduction

MCV is a novel human polyomavirus that has recently been discovered in Merkel cell carcinoma (MCC), a rare but highly aggressive skin cancer with neuroendocrine characteristics [1]. Several independent studies have confirmed that MCV is present in ∼80% of MCC tumors [[2] reviewed in [3]].

Polyomaviruses are small, non-enveloped viruses with a circular double-stranded DNA genome of about 5000 base pairs. The genome is divided into early and late coding regions separated by a noncoding regulatory region (NCRR). The NCRR contains the viral replication origin (Ori) and transcriptional regulatory elements/promoters that drive the bidirectional transcription of the early and late protein genes [3]. The MCV early region encodes three early proteins: large T antigen (LT), small T antigen (sT), and 57-kT antigen [4]. The LT protein mediates viral DNA replication [4]. The late region of the MCV genome encodes the capsid proteins VP1 and VP2.

During the normal viral life cycle, the polyomavirus genome is maintained as a circular dsDNA episome (also known as a viral minichromosome). In rare instances, the viral DNA can become integrated into the cellular DNA. Existing evidence suggests that nonproductive integration of MCV genomic DNA into the host cell DNA occurs early during MCC tumor development. It is thought that integration of MCV is a causal factor in a majority of MCC cases [3].

Simian virus 40 (SV40) is the most intensively studied member of the viral family *Polyomaviridae* and has long served as a model polyomavirus species. MCV appears to share a range of conserved sequences and biological functions with SV40. For example, the LT antigens of SV40 and MCV share a conserved LXCXE motif near the N-terminus of LT that participates in the inactivation of members of the pRb family of tumor suppressor proteins [5]. C-terminal portions of polyomavirus LT proteins also carry the conserved origin-binding domain (OBD) and helicase domains involved in unwinding of the viral origin of replication. Despite these clear functional similarities, it is important to note that MCV is not closely related to SV40 and recent studies have begun highlighting some differences in the way the two virus species interact with host cells [6]. SV40 was first discovered as an infectious contaminant in cell cultures used to grow poliovirus vaccine stocks. In contrast to SV40, MCV replicates very poorly in a wide range of cell lines [7], [8], [9]. Although transfected MCV genomic DNA appears to be maintained as a low copy number episome in some cell lines, MCV appears not to be able to establish a spreading infection in human tumor lines so far tested [10]. MCV can be propagated in an HEK-293 based line that stably expresses the MCV sT and LT proteins *in trans*, but even in this setting the infectious yield of MCV is orders of magnitude lower than SV40 [7].

The poor replication of MCV in cell culture is reminiscent of the biology of a different family of viruses, the *Papillomaviridae*. Papillomaviruses are strictly tropic for keratinocytes of the skin. Because the late phase of the viral life cycle is triggered by signals that are specific to differentiating keratinocytes, papillomaviruses, like MCV, generally cannot be propagated in conventional monolayer cell cultures. An additional parallel is that MCV, like human papillomaviruses (HPVs), is commonly shed from apparently healthy human skin surfaces. It has therefore been suggested that the MCV life cycle might be regulated by differentiation-dependent signals in epidermal cells.

In this study, we establish another possible parallel between the biology of MCV and HPVs. We demonstrate a functional interaction between the MCV LT antigen and the cellular bromodomain protein 4 (Brd4), a host cell factor that not only plays an important role in the papillomavirus life cycle but also associates with a number of other oncogenic viruses [11], [12], [13], [14].

Brd4 is a member of the BET family of proteins that harbor two bromodomains and an ET (extra-terminal) domain [15]. It plays a central role in cellular growth control, cell cycle progression, and cancer development [15], [16], [17], [18], [19], [20], [21], [22], [23], [24], [25]. Brd4 was previously shown to tether papillomavirus E2 protein/viral genomes to mitotic chromosomes, thus promoting faithful partitioning of replicated viral episomes to the nuclei of both daughter cells during mitosis [11]. It also plays an essential role in papillomavirus transcription [12], [26], [27], [28]. In addition, Brd4 has been suggested to contribute to papillomavirus DNA replication [26], although the underlying mechanism remains to be further investigated.

In this study, we demonstrate that Brd4 interacts with MCV LT and may contribute to MCV DNA replication by recruiting the cellular protein replication factor C (RFC). Our study thus brings new mechanistic insight into MCV replication, demonstrating a novel function of Brd4 in viral replication and the MCV life cycle.

## Results

### Human Brd4 protein interacts with MCV LT

**Figure 1:**
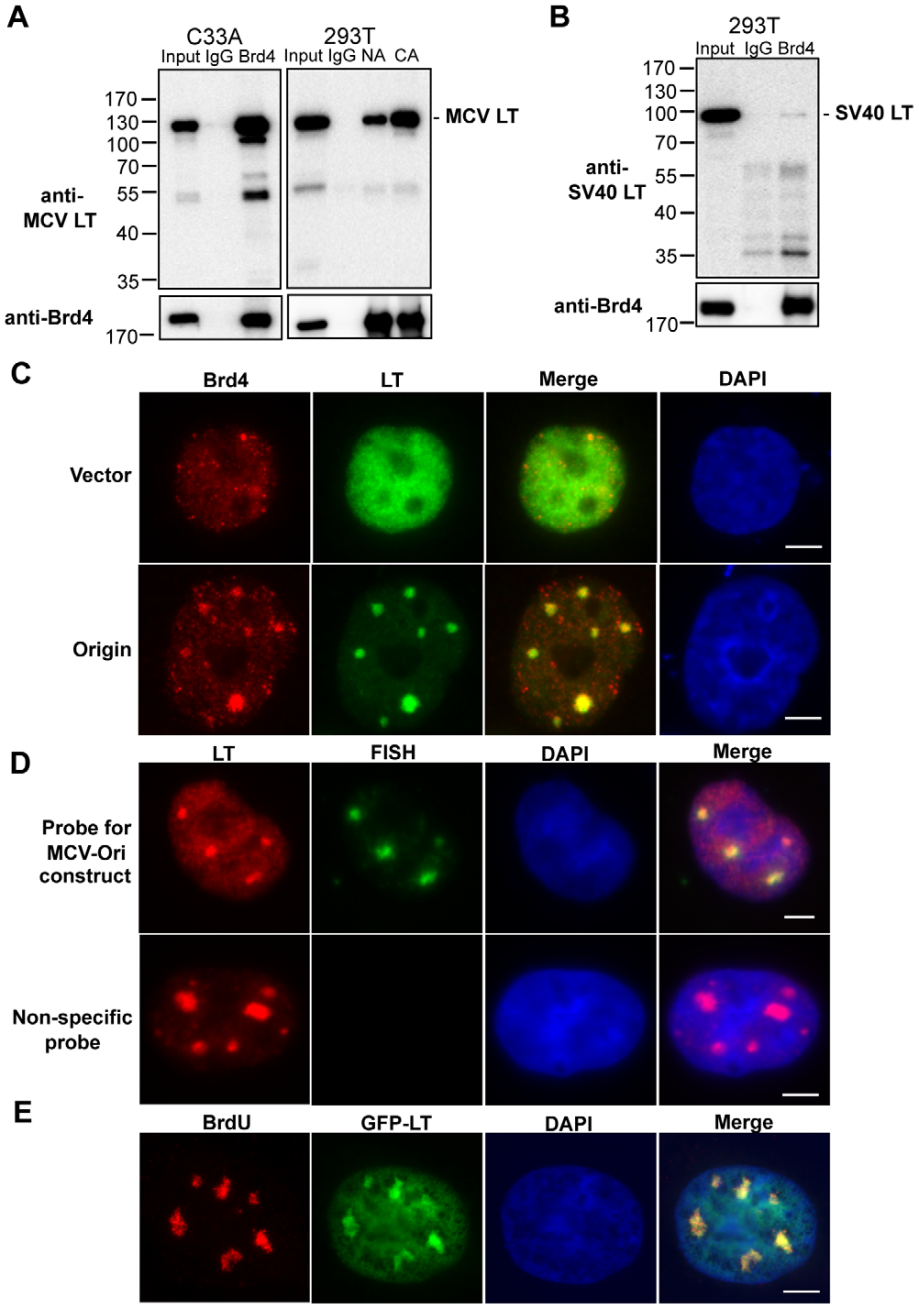
Human Brd4 interacts with MCV LT. **A**. MCV LT Co-IP with Brd4. C33A cells were transfected with pcDNA4C-MCV LT. Nuclear extracts IPed with normal rabbit IgG (IgG) or the polyclonal Brd4NA antibody (Brd4) were immunoblotted for MCV LT and Brd4. This experiment was repeated three times with similar results. 293T cells were transfected with pADL* (an LT expression plasmid). Nuclear extracts were IPed using normal rabbit IgG (IgG), Brd4 N-terminal antibody (NA) or Brd4 C-terminal antibody (CA). 20% of the Co-IP samples and 20 µg nuclear extracts (input) were immunoblotted for MCV LT and Brd4. **B**. SV40 LT interacts weakly with human Brd4. 293T nuclear extracts IPed with normal rabbit IgG (IgG) or Brd4CA antibody (Brd4) were immunoblotted for SV40 LT and Brd4. **C**. Co-localization of Brd4 and MCV LT. C33A cells were co-transfected with pcDNA4C-MCV LT and either pcDNA4C-MCV Ori (Origin) or pcDNA4C (Vector). Cells were double-stained with Brd4 CA (Red) and MCV LT antibody (Green). Cells were also counter-stained with DAPI. Bar, 5 µm. **D**. MCV Ori DNA is detected in the MCV LT foci. C33A cells were co-transfected with pADL* and pcDNA4C-MCV Ori. A specific probe recognizing pcDNA4C-MCV Ori and a non-specific probe recognizing the HPV genome were used in Immuno-FISH analysis. Cells were stained with MCV LT antibody (Red) and DNA probes were stained with fluorescein avidin DN (Green). Bar, 5 µm. **E**. C33A cells were co-transfected with pcDNA4C-MCV LT, pEGFP-MCV LT and pcDNA4C-MCV Ori. pEGFP-MCV LT was used to mark the MCV LT localization. Cells were pulsed with 10 µM BrdU for 20 min and stained with anti-BrdU antibody (Red). Cells were also counter-stained with DAPI. Bar, 5 µm. All experiments have been repeated more than 3 times with similar results.

In a proteomic study using mass spectrometry to identify Brd4 interacting proteins in 293T cells, we isolated four different peptides that match the SV40 LT protein, which is stably expressed by this line (Fig. S1). We therefore set out to investigate whether SV40 LT binds Brd4. Based on the intriguing potential similarities between the MCV life cycle and the HPV life cycle, in which Brd4 plays an important role, we also chose to test the interaction between MCV LT and Brd4. Although SV40 LT appeared to bind Brd4 very weakly in 293T cells, MCV LT was efficiently co-immunoprecipitated (Co-IPed) with Brd4 (Fig. 1A and 1B). In C33A cells, nearly 40% of the MCV LT was Co-IPed with a polyclonal Brd4 antibody. This result was confirmed in 293T cells using polyclonal Brd4 NA antibody (targeting Brd4 aa 156–284) or Brd4 CA antibody (recognizing aa 1313–1362), which could Co-IP about 20% of the total MCV LT in this cell type (Fig. 1A). In control cells transfected with MCV sT, Brd4 did not pull down any proteins with the same size as MCV LT detectable on T antigen Western blots (Fig. S2A). Using a reciprocal binding assay, we also confirmed that bacterially expressed GST-MCV LT could pull down Brd4, while a much higher level of GST alone showed no binding (Fig. S2B). To determine the stability of the Brd4-MCV LT interaction, the Brd4-MCV LT immuno-complexes isolated on Protein A sepharose were incubated with buffer containing increasing salt concentrations (Fig. S2C). No change was detected in the MCV LT association with Brd4 immuno-complexes in the presence of 300 mM sodium chloride, and it was only slightly dissociated from the complex when the sodium chloride concentration was increased to 500 mM and 1000 mM. These data demonstrate the specific interaction between Brd4 and MCV LT.

To further investigate the biological impact of the Brd4 and MCV LT interaction, we performed immunofluorescent staining (IF) to examine the localization of Brd4 and MCV LT in cells. In C33A cells, exogenously expressed MCV LT shows diffuse nuclear staining (Fig. 1C). However, in cells co-transfected with the LT expression plasmid and a separate plasmid carrying the 97 base pair (bp) MCV replication Ori (pcDNA4C-MCV Ori), which does not include the MCV early gene promoter (see details in Text S1), 28.9±2.3% (n = 3) of the LT-positive cells showed recruitment of MCV LT to punctate nuclear foci (Fig. 1C). More than 100 cells were counted in each sample. This result suggests that the MCV LT interaction with the viral Ori mediates trafficking of the resulting DNA replication complex to specific subnuclear locations (Fig. 1C). In cells transfected with MCV LT and MCV Ori-less control vector, endogenous Brd4 signal was spread throughout the nucleus, with some signals enriched in small punctate nuclear foci. This Brd4 staining pattern appears more diffuse than in the normal cells where Brd4 localizes to punctate chromatin foci, suggesting that MCV LT may induce the re-localization of Brd4. Remarkably, in cells co-transfected with LT and the MCV Ori^+^ plasmid, Brd4 was recruited to large LT-positive foci (Fig. 1C). A similar result was observed in U2OS cells (data not shown).

Immunostaining-fluorescence in situ hybridization (Immuno-FISH) showed that, as expected, the MCV Ori DNA is specifically detected in the MCV LT foci (Fig. 1D). Metabolic labeling with 5-bromo-2′-deoxyuridine (BrdU) also revealed that these MCV Ori DNA foci contain actively replicating DNA (Fig. 1E). As we show below, other replication factors are also recruited to these foci, suggesting that these Ori-containing nuclear foci represent the MCV replication complex. Taken together, the results suggest that Brd4 is recruited to the MCV replication complex through interaction with MCV LT.

### Brd4 is important for MCV DNA replication

**Figure 2:**
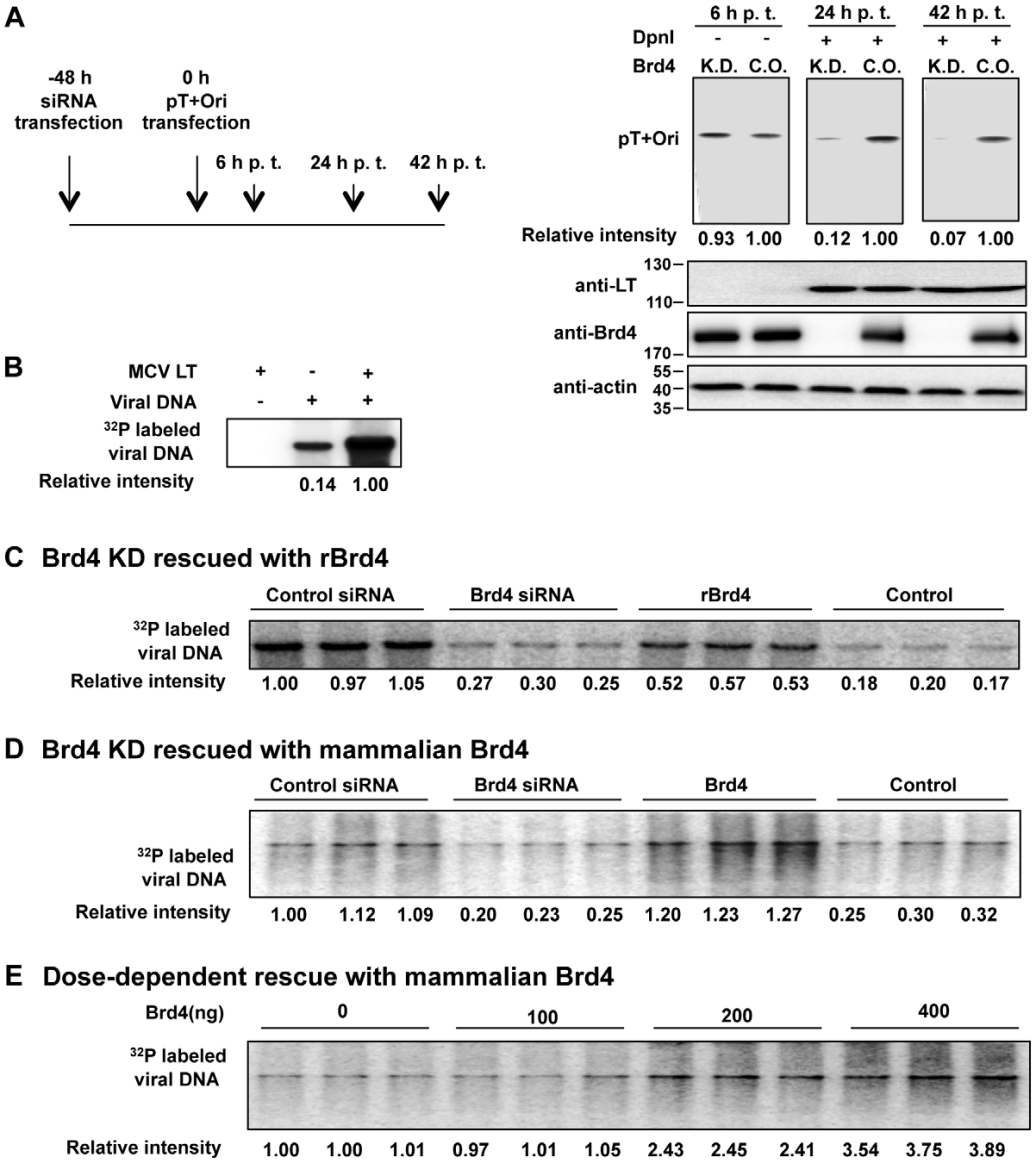
Brd4 is important for MCV DNA replication. **A**. Brd4 knockdown inhibits MCV replication *in vivo*. C33A cells were transfected with either a siRNA targeting Brd4 (K.D.) or a non-targeting siRNA control (C.O.). Forty-eight h later, cells were transfected with pT+Ori and this time was set as 0 h. Total DNA was extracted at 6, 24 and 42 h p.t.; 2 µ*g* of the DNA samples from 6 h p.t. were digested with EcoRI and plasmid DNA was detected by Southern blotting. 10 µ*g* of the DNA samples from 24 and 42 h p.t. were digested with both EcoRI and DpnI to specifically detect replicated plasmid. Protein extracts were immunoblotted for MCV LT, Brd4 and actin. **B**. MCV LT-dependent *in vitro* replication of MCV genome. *In vitro* MCV replication was performed using full length MCV genomic DNA and cell extracts prepared from 293T cells transfected with either pcDNA4C-MCV LT or pcDNA4C. **C**. Brd4 knockdown inhibits viral DNA synthesis *in vitro* and the inhibition can be rescued by recombinant Brd4. 293T cells were transfected with a Brd4 siRNA or a control siRNA. At 40 h p.t., cells were re-transfected with pcDNA4C-MCV LT. Cell extracts were prepared at 88 h p.t. and used for *in vitro* replication of MCV DNA. In the “rBrd4” condition, 3 µg His-Brd4 purified from insect cells using nickel resin was added to the Brd4 knockdown extract prior to performing the replication assay. In the “Control” condition, an equal amount of nonspecific proteins eluted from the nickel resin incubated with insect cells carrying wild-type baculovirus were used. All reactions were performed in triplicates. Immunoblots of cell extracts used in the assay and His-Brd4 purified from insect cells are shown in Fig. S3B and S3C. **D**. Brd4 knockdown inhibits MCV DNA replication *in vitro* and the inhibition can be rescued by Brd4 purified from mammalian cells. Extracts from cells transfected with a Brd4 siRNA and pcDNA4C-MCV LT as described in **C** were used in the *in vitro* replication assay. In the “Brd4” condition, 400 ng Brd4 purified from 293T cells was added to the Brd4 knockdown extract prior to performing the replication assay. In the “Control” condition, an equal amount of nonspecific proteins isolated from the vector control cells were used. All reactions were performed in triplicates. Coomassie Brilliant Blue staining of Brd4 purified from 293T cells are shown in Fig. S3E. **E**. Dose-dependent rescue of *in vitro* viral replication by the purified Brd4 protein. Extracts from cells transfected with a Brd4 siRNA and pcDNA4C-MCV LT as described in **C** were used in the *in vitro* replication assay. Increasing amounts of purified Brd4 was added to the reactions. All reactions were performed in triplicates.

The data in Fig. 1 suggested that Brd4 is recruited to the MCV replication complex to contribute to viral DNA replication. To investigate the role of Brd4 in MCV replication, we examined how Brd4 knockdown affects viral DNA replication in cells transfected with pT+Ori, a self-replicating plasmid in which MCV LT and sT are expressed from the MCV NCRR (see details in Text S1). Analysis of DNA samples harvested at 6 h after pT+Ori transfection showed that roughly the same amount of DNA was transfected into Brd4 knockdown and control cells (Fig. 2A). Plasmid replication was monitored at 24 and 42 h post transfection (h p.t.) by treating the DNA samples with DpnI to eliminate transfected DNA. Southern blotting revealed that the replication of pT+Ori was inhibited in the Brd4 siRNA-treated cells by 88–93% (Fig. 2A, compare K.D. with C. O. p<0.001 at both 24 and 42 h p.t. n = 3). Similar results were obtained from several Southern blotting analyses using either pT+Ori or recombinant full-length wild-type MCV genomic DNA reconstituted by intramolecular re-ligation (data not shown). IF analysis also showed that Brd4 knockdown abrogated the MCV replication complex formation (Fig. S3A). MCV LT expression from the MCV NCRR was not affected by Brd4 knockdown, indicating that Brd4 is not directly involved in MCV transcription (Fig. 2A). These results suggest that Brd4 is critical for supporting MCV DNA replication in the host cells.

Brd4 is known to play a critical role in cellular transcription and growth; therefore Brd4 knockdown might indirectly inhibit viral DNA replication by blocking the transcription of important replication factors or affecting the cell cycle progression. To investigate the possibility of a direct role of Brd4 in viral replication, we examined the Brd4 function in MCV replication using a cell-free *in vitro* replication assay [29]. We first established that lysates of cells expressing MCV LT could efficiently stimulate MCV replication *in vitro* (Fig. 2B). Cells were then transfected with an MCV LT expression construct and either Brd4 siRNA or control siRNA. Western blotting showed that Brd4 was efficiently knocked down in Brd4 siRNA-treated cells without negatively affecting MCV LT expression (Fig. S3B). Despite the slightly greater abundance of LT in the Brd4 siRNA-treated cell lysate, MCV replication was inhibited by 67–80% in the Brd4 knockdown cell lysate (Fig. 2C, p<0.001, n = 3). Brd4 knockdown did not achieve 100% inhibition of the *in vitro* MCV replication likely because, as observed in Fig. 2B, a low level of the MCV DNA was replicated independent of MCV LT and therefore might not be affected by Brd4 knockdown. More importantly, addition of recombinant insect cell-derived Brd4 protein to the Brd4 knockdown cell lysate partially restored the MCV replication (Fig. 2C, p<0.001, n = 3, Fig. S3C). Furthermore, a dose-dependent rescue of the MCV replication was observed with increasing amounts of recombinant Brd4 (Fig. S3D). In addition, recombinant Brd4 expressed in 293T cells and purified to near homogeneity (Fig. S3E) could also rescue the MCV replication in a dose-dependent manner (Fig. 2D and 2E). This dose-dependent rescue of MCV replication in the Brd4 knockdown cell lysate with recombinant Brd4 suggests that MCV replication is dependent on the recombinant Brd4 protein added into the reaction. The fact that larger amount of recombinant Brd4 gave better rescue could be because not all purified Brd4 proteins are as appropriately folded as endogenous Brd4 protein. In addition, the recombinant Brd4 protein samples also contain shorter Brd4 proteolytic cleavage products (Fig. S3), which may function as DNIs to reduce the activity of full-length Brd4 protein such that a higher concentration of the protein is need to achieve efficient rescue. In summary, the results demonstrate that Brd4 facilitates MCV DNA replication both in intact cells and in cell free extracts.

### Mapping of Brd4-MCV LT interaction sites

**Figure 3:**
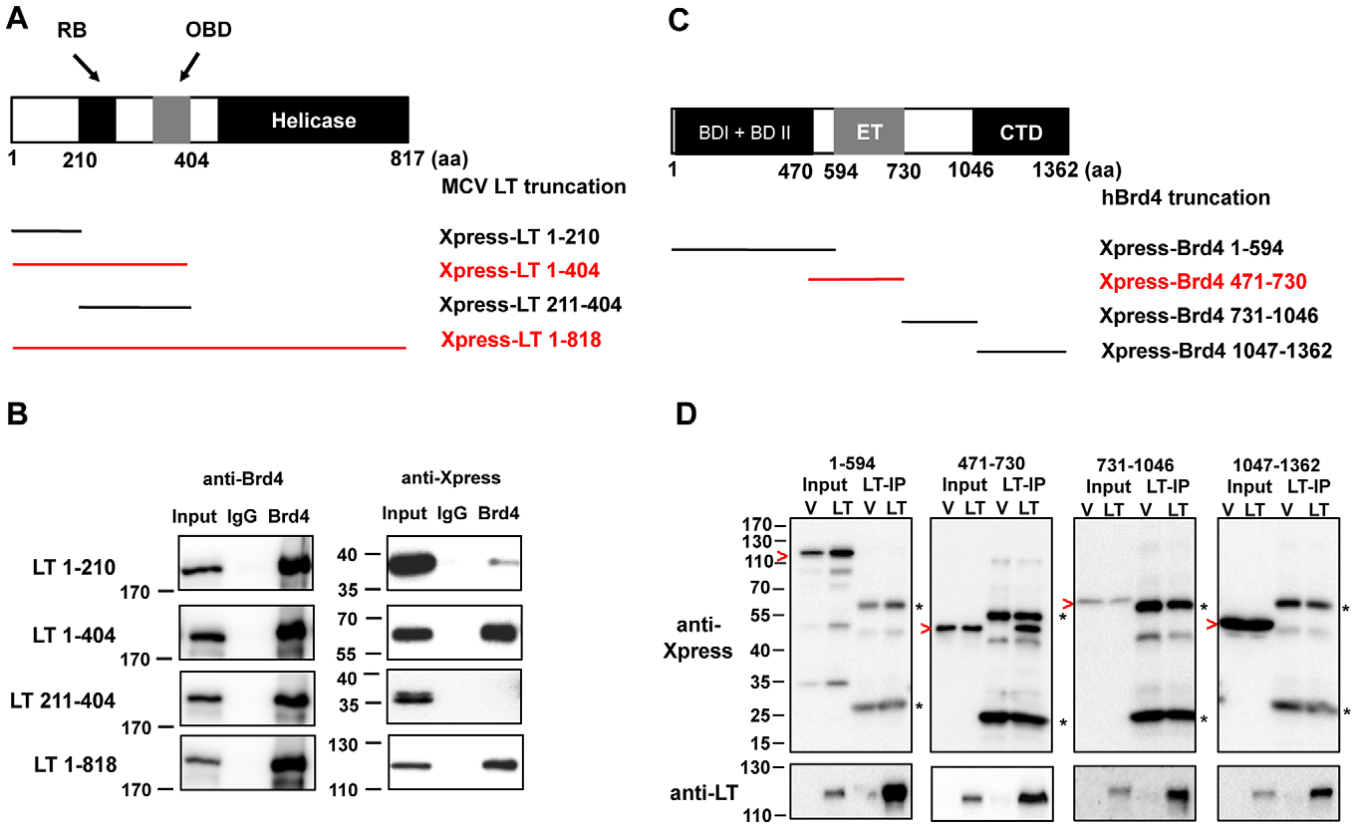
Mapping of the MCV LT and Brd4 interaction sites. **A**. Schematic diagrams of full-length MCV LT protein and truncation mutants used for mapping. All MCV LT fragments were expressed from a pcDNA4C plasmid encoding an Xpress epitope tag. **B**. Mapping the Brd4 binding site on MCV LT. 293T cells were transfected with full-length MCV LT or a truncation mutant construct. Nuclear extracts Co-IPed with normal rabbit IgG (IgG) or a Brd4 antibody (Brd4) were immunoblotted with Xpress (LT) or Brd4 antibodies. **C**. Schematic diagrams of full-length human Brd4 protein and truncation mutants used for mapping. All Brd4 fragments were expressed from a pcDNA4C plasmid as Xpress tagged proteins. **D**. Mapping the MCV LT binding site on Brd4. 293T cells were co-transfected with a Brd4 mutant construct and either the FLAG-tagged pOZN vector (V) or pOZN-MCV LT (LT). Nuclear extracts IPed with anti-FLAG beads were immunoblotted with Xpress (Brd4) or LT antibodies. Arrowheads mark the positions of the Xpress tagged Brd4 mutants. Asterisks to the right of the blots mark the positions of IP antibody heavy and light chains. For all IPs, 20 µg nuclear protein input and 20% of Co-IPed samples were analyzed.

To further understand how Brd4 contributes to MCV DNA replication, we examined the molecular details of the Brd4 and MCV LT interaction. The MCV LT protein was truncated into different fragments based on its putative functional domains (Fig. 3A and data not shown). These truncation mutants were tested for the ability to interact with endogenous Brd4 by Co-IP. Among all of the mutants tested, only MCV LT 1-404 was most efficiently Co-IPed with endogenous Brd4, while the shorter LT truncation mutant 1–210 and 211–404 showed nearly no apparent interaction with Brd4 (Fig. 3B and data not shown). These data demonstrate that the intact N-terminal half of MCV LT is important for binding Brd4, but the helicase domain is dispensable.

To map the MCV LT binding site on Brd4, the Brd4 protein was truncated into different fragments based on its known functional domains (Fig. 3C) [30]. 293T cells were co-transfected with a construct encoding an Xpress tagged Brd4 truncation mutant and either the empty pOZN control vector or Flag-tagged pOZN-MCV LT. Anti-Flag immunoprecipitation (IP) showed that Brd4 471-730 fragment could be efficiently Co-IPed with MCV LT, while the rest of the Brd4 mutants show negative binding (Fig. 3D). The MCV LT binding site is therefore mapped to the Brd4 471-730 region, which includes the ET domain.

To confirm this mapping result, GST tagged Brd4 471-730 was tested for binding to *in vitro* translated MCV LT (Fig. S4). MCV LT was pulled-down by GST-Brd4 471-730 but not GST alone (Fig. S4). This result confirmed the *in vivo* mapping results and demonstrated that Brd4 471-730 is directly involved in binding MCV LT.

### Brd4 471-730 functions as a dominant negative inhibitor (DNI) of the Brd4 and MCV LT interaction

**Figure 4:**
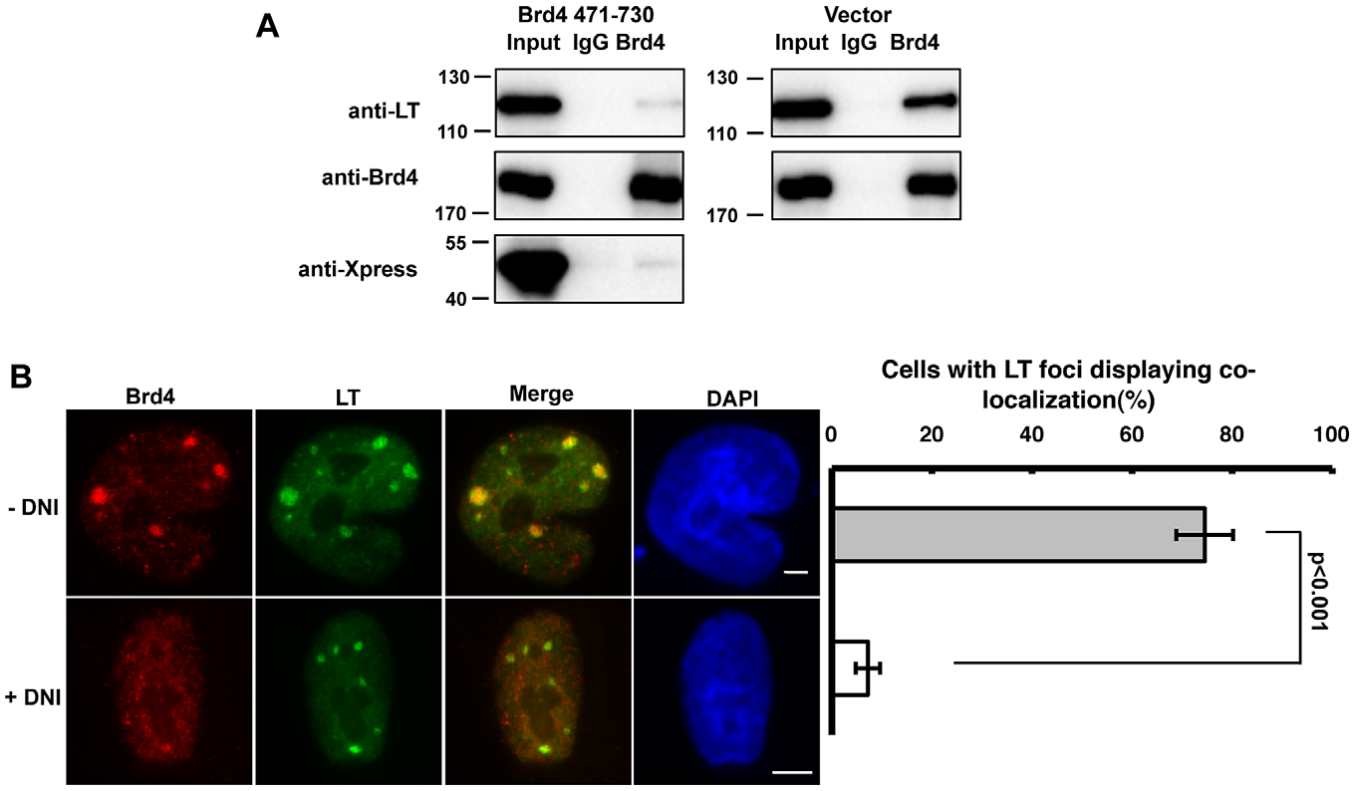
Brd4 471-730 functions as a DNI of the MCV LT and Brd4 interaction. **A**. Brd4 471-730 inhibits MCV LT and Brd4 Co-IP. 293T cells were co-transfected with pcDNA4C-MCV LT together with a pCDNA4C plasmid encoding Xpress-tagged Brd4 471-730 or the empty vector. Nuclear extracts IPed using either normal rabbit IgG or Brd4 NA were immunoblotted using an antibody against LT, or against the Xpress epitope tag carried by the Brd4 truncation mutant, or Brd4 CA antibody. **B**. The DNI inhibits Brd4 and MCV LT punctate co-localization in cells. C33A cells co-transfected with a MCV LT construct and either pcDNA4C-MCV Ori (−DNI) or pcDNA4C-MCV Ori-DNI (+DNI) were stained with Brd4 CA (Red) and MCV LT antibody (Green). Bar, 5 µm. Mean and standard deviation of the percentage of cells with LT foci displaying Brd4 and LT co-localization in punctate dots were calculated from three independent experiments. More than 100 cells were counted in each sample.

Because Brd4 471-730 can bind MCV LT in cells (Fig. 3), we tested if it could function as a dominant negative inhibitor (DNI) to block the MCV LT interaction with endogenous Brd4. Brd4 and MCV LT Co-IP was performed using cells co-expressing MCV LT and the Brd4 471-730 fragment. Brd4 NA antibody, which does not recognize the Brd4 471-730 fragment, could efficiently Co-IP MCV LT in the presence of the vector control, but not in cells co-expressing Brd4 471-730 (Fig. 4A). In addition, Brd4 471-730 could inhibit the Brd4 and MCV LT Co-IP in a dose-dependent manner (Fig. S5). This establishes that Brd4 471-730 functions as a DNI to disrupt the MCV LT and Brd4 interaction.

We also examined how Brd4 471-730 could affect Brd4 and MCV LT co-localization in the viral replication foci. To ensure that cells carrying the MCV replication Ori also express Brd4 471-730, we generated the pcDNA4C-MCV Ori-DNI construct (MCV Ori-DNI), in which the DNI expression cassette, including a CMV promoter, Xpress-tagged DNI open reading frame and a polyadenylation signal, was inserted into the pcDNA4c-MCV Ori plasmid (see details in Text S1). Brd4 471-730 expression from this construct was detected by Western blotting and its nuclear localization was confirmed by IF (Fig. S6). In cells co-transfected with pcDNA4C-MCV LT and the MCV Ori construct, more than 75% of the cells with LT foci displayed Brd4 and LT co-localization (Fig. 4B, −DNI, n = 3). However, in the majority of cells co-transfected with pcDNA4C-MCVLT and the MCV Ori-DNI construct, Brd4 was no longer recruited to the MCV LT replication foci (Fig. 4B, +DNI, n = 3). More than 100 cells were counted for each sample and the quantification results are summarized in Fig. 4B. These results show that Brd4 471-730 functions as a DNI to inhibit the recruitment of Brd4 to MCV LT/viral Ori replication foci.

### Brd4 471-730 inhibits MCV DNA replication

**Figure 5:**
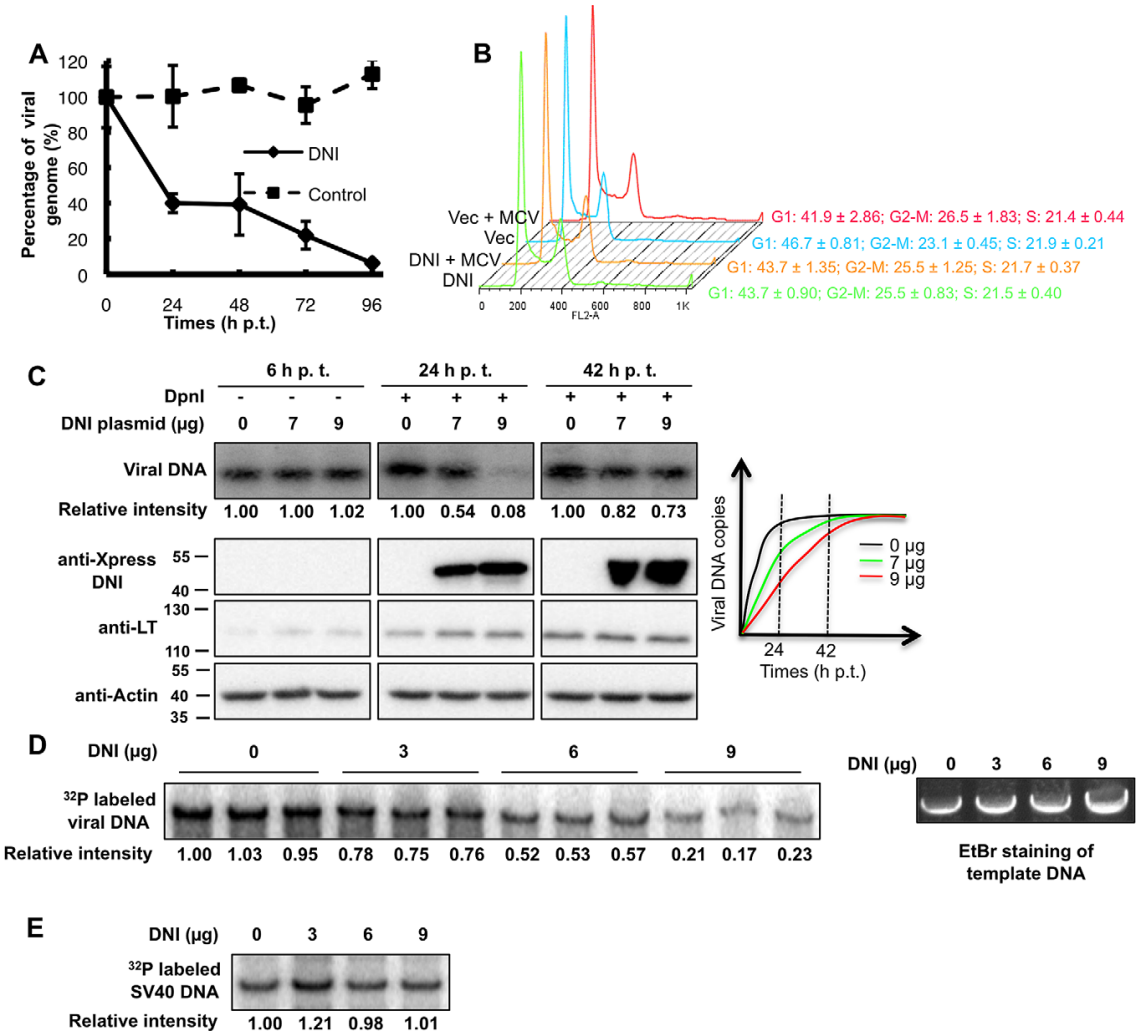
The DNI inhibits MCV DNA replication. **A**. DNI expression reduces MCV genome replication in an MCV sT/LT stable cell line. 293-4T cells were transfected with the MCV genome. At 84 h p.t., cells were re-transfected with either pcDNA4C-Brd4 471-730 (DNI) or pcDNA4C (control). The time for the second transfection was set as 0 hr. Viral genomes present in cellular DNA extracted at different time points were quantified using qPCR. Viral genome copies were normalized to beta-actin DNA and presented as a percentage of the viral genome detected at 0 hr. Mean and standard deviation were calculated from three independent experiments. **B**. FACS cell cycle analysis. 293T cells were transfected with pcDNA4C (Vec) or pcDNA4C-Brd4 471-730 (DNI) either alone or together with re-ligated MCV genome (MCV) as indicated. At 48 h p.t., cells were fixed and subjected to FACS analysis. **C**. The DNI inhibits autonomous MCV replication. 293T cells were co-transfected with 1 µg MCV genome and either 0, 7, or 9 µg of pcDNA4C-Brd4 471-730 (DNI). 2 µg of cellular DNA from 6 h p.t. was digested with EcoRI to show that equal amount of viral genomes were transfected into cells. 10 µg of DNA extracted from 24 and 42 h p.t. was digested with EcoRI and DpnI to detect the replicated viral DNA. The viral DNA was analyzed using Southern blotting. Intensities of autoradiography signal were analyzed using ImageJ and normalized to the value obtained with 0 µg of DNI at each time point. Protein extracts were immunoblotted for Xpress-DNI, MCV LT and actin. A schematic diagram of MCV replication time-course is also shown. **D**. The DNI inhibits viral DNA synthesis *in vitro*. 293T cells were co-transfected with pcDNA4C-MCV LT and pcDNA4C-Brd4. Cellular extracts were supplemented with increasing amount of recombinant DNI and used in the *in vitro* MCV replication assay. All reactions were performed in triplicates. DNA from the same reactions omitting [α-^32^P] dCTP and creatine kinase were resolved on an agarose gel and stained with ethidium bromide. **E**. The DNI does not inhibit SV40 replication *in vitro*. *In vitro* replication using 293T cell extracts and pEGFP-C1 (carrying SV40 Ori) as template was performed as described in **D**. Increasing amount of recombinant DNI was added to the *in vitro* replication assays.

Because Brd4 471-730 can competitively bind to MCV LT to prevent its interaction with endogenous Brd4, it provides a useful tool to investigate the role of the Brd4-MCV LT interaction in MCV genome replication. In an initial set of experiments, 293-4T cell, which stably express MCV LT and sT, were transfected with the re-ligated MCV genome to allow establishment of replicating viral episomes. The 293-4T with MCV episome cells were super-transfected with either the DNI construct or a control plasmid, and the viral genome was quantified using qPCR (Fig. 5A). While the average episome content in the control cells remained at a similar level throughout analysis, DNI co-expression reduced the episome copy number by ∼60% within the first 24 h and ∼94% after 4 days (Fig. 5A). FACS analysis showed that cells transfected with DNI alone or both DNI and MCV genome displayed similar cell cycle profile compared to vector controls (Fig. 5B, n = 3), suggesting that the reduction of episome copy number caused by DNI was unlikely to have been due to an indirect effect attributable to cell cycle perturbation. This result shows that the DNI inhibits LT-mediated MCV genome replication in an MCV sT/LT stable cell line.

We also investigated if Brd4 471-730 could interfere with MCV viral replication during the autonomous establishment of MCV infection. 293T cells were co-transfected with 1 µg of MCV genome and either 0, 7, or 9 µg of pcDNA4C-Brd4 471-730 (Fig. 5C). DNA extracted 6 h later was analyzed without DpnI digestion to show that an equal amount of viral genomes were transfected into cells (Fig. 5C). DNA samples extracted at later time points were digested with DpnI to specifically detect the replicated viral DNA. Although DpnI-resistant viral DNA increased dramatically from 6 to 42 h in the absence of DNI expression, co-expression of the DNI inhibited the viral DNA replication by 46%–92% at 24 h depending on the amount of DNI (Fig. 5C). The inhibition was most clearly observed at 24 h with nearly no detectable newly synthesized viral DNA in cells transfected with 9 µg of the DNI construct (Fig. 5C). A similar inhibition time-course was observed in several different experiments and also in C33A cells (data not shown). The lower level of DNI inhibition at 42 h could be because the overall viral DNA replication reached a higher level at this time point as indicated by the increased MCV LT level (Fig. 5C). In addition, the reduced DNI effect at 42 h could mean that the DNI could only reduce the kinetics of the viral replication so that the replicated MCV genome reached the plateau at a later time than in the control cells (Fig. 5C, a schematic diagram in the right panel). In line with this notion, BrdU pulse labeling of the replicating viral DNA also showed that, in cells expressing DNI, the kinetics of viral DNA replication was delayed (Fig. S7A). The dose-dependent inhibition of viral replication by the DNI is consistent with the fact that it inhibits the Brd4 and MCV LT interaction more efficiently at higher concentration (Fig. S5). Notably, the MCV LT expression from the viral genome was not affected by DNI expression, providing additional evidence to support that Brd4 is not directly involved in MCV transcription regulation (Fig. 5C). To rule out the possibility that the DNI may indirectly inhibit viral replication by affecting cellular replication or growth, we showed that DNI expression did not interfere with cellular DNA replication (Fig. S7B). We also analyzed the growth rate of cells expressing MCV LT, Brd4 471-730 or both. Our analysis showed that Brd4 471-730 expression did not introduce cellular toxicity, but rather slightly ameliorated the growth-inhibitory effect of MCV LT (Fig. S8). Together, these results indicate that Brd4 471-730 functions to directly inhibit MCV DNA replication in cells by breaking the Brd4 and MCV LT interaction.

To confirm that Brd4 471-730 can directly inhibit MCV viral replication, we tested recombinant Brd4 471-730 in the *in vitro* MCV DNA replication assay. Addition of increasing amounts of recombinant Brd4 471-730 caused up to 77–83% reduction of the newly synthesized MCV DNA (Fig. 5D, p<0.001, n = 3). DNA samples from the reactions omitting [α-^32^P] dCTP and creatine kinase showed that the same amount of DNA templates was used in each reaction (Fig. 5D). In accordance with the observation that Brd4 does not show strong interaction with SV40 LT (Fig. 1B), recombinant Brd4 471-730 did not significantly inhibit SV40 replication *in vitro* (Fig. 5E). Together these data strongly suggest that, through disrupting the Brd4 and MCV LT interaction, Brd4 471-730 can efficiently inhibit MCV DNA replication *in vitro*. Thus, both *in vivo* and *in vitro* data demonstrate that the Brd4 and MCV LT interaction is important for MCV replication.

### Releasing endogenous Brd4 from chromatin enhances MCV DNA replication

**Figure 6:**
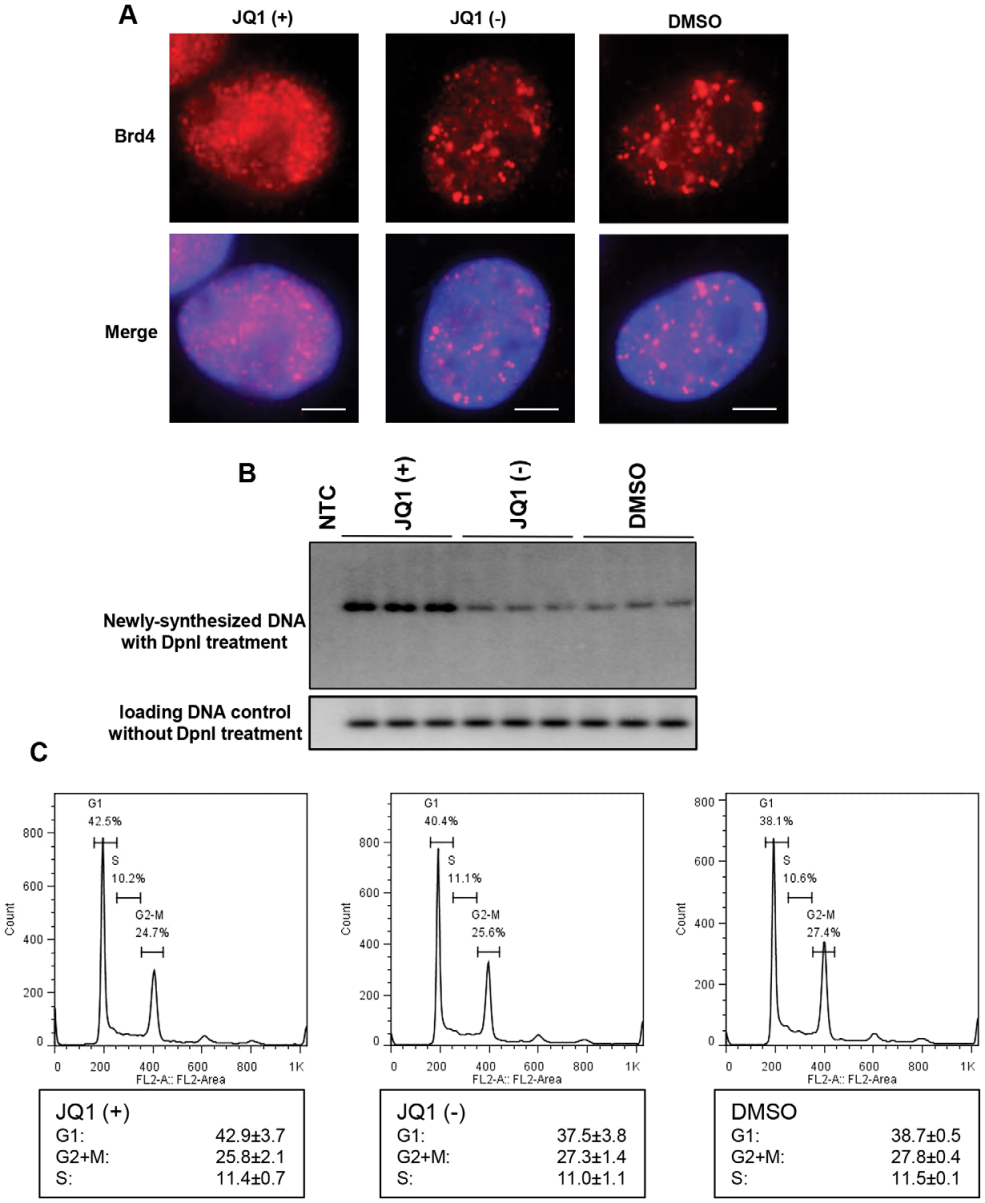
JQ1(+) treatment increases the MCV replication. **A**. JQ1(+) releases Brd4 from chromatin. C33A cells were treated with 300 nM JQ1(+), JQ1(−) or DMSO for 24 h. Cells were fixed and stained with Brd4 antibody and DAPI. **B**. JQ1(+) treatment increases the MCV replication. C33A cells were transfected with pT+Ori and split into 3 dishes. At 24 h p.t., cells were treated with 300 nM JQ1(+), JQ1(−) or DMSO. Total DNA was extracted at 48 h p.t., and 20 µg of XhoI/DpnI-digested DNA was loaded in each lane for Southern blotting to detect the replicated plasmid. 1.5 µg total DNA samples that have been digested with XhoI but not with DpnI were also analyzed in Southern-blotting to show equal loading of DNA. NTC, no transfection control. All reactions were performed in triplicates. **C**. JQ1(+) treatment does not affect cell cycle. C33A cells were cultured for 12 h and treated with 300 nM JQ1(+), JQ1(−) or DMSO. Cells were cultured for another 24 h and fixed for FACS cell cycle analysis.

Most of Brd4's function in regulating cellular growth and cell cycle progression has been linked to its ability to bind to acetylated histones on chromatin. Since MCV LT/Ori expression appears to induce the re-localization of Brd4 to viral replication foci, it is conceivable that MCV LT competes with host chromatin for access to Brd4. To determine if the role of Brd4 in MCV DNA replication could be uncoupled from its cell cycle and transcriptional regulation function, we tested the effect of a specific inhibitor of the Brd4-chromatin interaction. The chemical compound JQ1(+) has been established as a highly selective inhibitor of Brd4 that prevents its binding to the tetra-acetylated histone H4 peptide [31]. We verified that JQ1(+) efficiently dissociated Brd4 from punctate chromatin foci into diffuse nuclear localization patterns in untransfected C33A cells, while the cells treated with the inactive stereoisomer JQ1(−) or DMSO vehicle control showed normal punctate Brd4 pattern (Fig. 6A). The reorganization of Brd4 in JQ1(+) treated cells likely reflects Brd4 displacement from chromatin foci. Interestingly, treatment with 300 nM JQ1(+) led to increased pT+Ori MCV DNA replication compared to cells treated with either 300 nM JQ1(−) or DMSO (Fig. 6B). FACS analysis showed that cells treated with JQ1(+), JQ1(−) or DMSO display a similar cell cycle profile (Fig. 6C, n = 3), suggesting that the enhanced MCV DNA replication is not caused by a cell cycle effect associated with JQ1(+) treatment. These experiments suggest that freeing Brd4 from chromatin enhances MCV DNA replication, presumably by increasing the availability of Brd4 for assembly of MCV LT/Ori replication complexes.

### MCV LT mediates unwinding of viral Ori DNA

DNA replication requires the concerted action of many cellular factors. Although the results above establish that Brd4 is important for MCV replication, the underlying mechanisms are unclear. To begin to address this issue, we examined the recruitment of various cellular factors to MCV LT/Ori replication foci.

**Figure 7:**
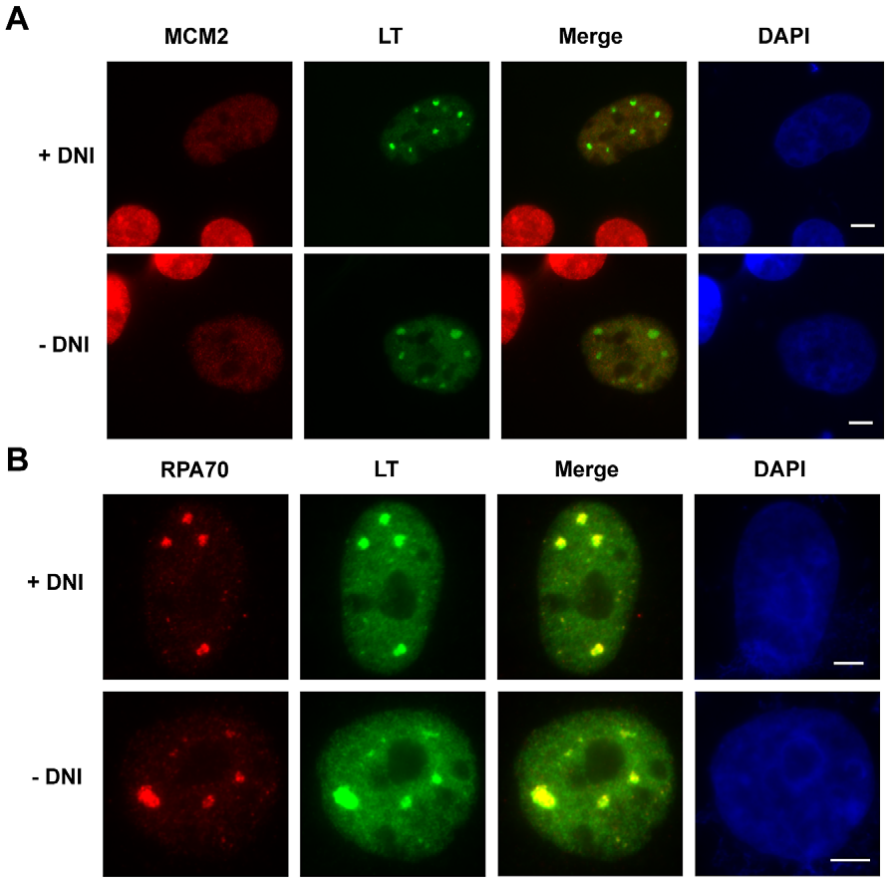
The MCV replication complex. **A**. The host helicase MCM2 does not co-localize with MCV LT in the MCV replication foci. C33A cells were co-transfected with pcDNA4C-MCV LT and either pcDNA4C-MCV Ori (−DNI) or pcDNA4C-MCV Ori-DNI (+DNI). Cells were stained using antibodies for MCM2 (Red) and MCV LT (Green). Bar, 5 µm. **B**. The DNI does not inhibit viral Ori unwinding. C33A cells transfected as in **A** were stained using antibodies for RPA70 (Red) and MCV LT (Green). Bar, 5 µm.

Origin activation during DNA replication involves unwinding of the parental DNA duplex by the replicative helicase and assembly of replisomes around the activated helicase. Eukaryotic viral helicases, such as the SV40 LT and the papillomavirus E1 protein, form double hexamers at the viral origin to induce origin melting and processive DNA unwinding, while recruiting other cellular DNA replication components to the active replication forks. In host DNA replication, the minichromosome maintenance (MCM2-7) complex functions as the eukaryotic replicative helicase [32]. To rule out the possibility that the MCV LT associated nuclear foci are the sites of host DNA replication, we stained the MCV LT and Ori positive cells with antibodies against MCV LT and MCM2, a subunit of the MCM complex. While MCV LT staining detected the characteristic punctate nuclear foci, MCM2 IF showed a clearly diffuse signal across the nucleus without specific colocalization with the MCV LT foci (Fig. 7A). The MCM2 signal was also dimmer in the MCV LT and Ori positive cells. The same pattern was observed in cells expressing the DNI (Fig. 7A). The results suggest that the MCV LT/Ori replication foci are not host DNA replication hot spots but rather the viral DNA replication sites. In this model, MCV LT (but not the host helicase MCM2) is recruited to these sites, where it likely functions as a helicase that unwinds the viral DNA in preparation for replication. The lack of MCM2 accumulation at the MCV LT/Ori foci also suggests that these foci are not likely formed by non-specific aggregation of cellular replication factors.

After the DNA helicase initiates DNA replication by unwinding the double stranded DNA, single-stranded DNA (ssDNA) is stabilized by members of the replication protein A (RPA) family [33]. RPA subunits assemble into the nascent replication complex to prevent complementary ssDNA from reannealing or from forming secondary structures that would interfere with DNA processing [33]. RPA is therefore essential for accurate and processive DNA synthesis. RPA was first identified as an indispensable component of SV40 LT-mediated DNA replication *in vitro* [34]. As expected, IF analysis showed that RPA70 is specifically recruited to the MCV LT/Ori foci (Fig. 7B). In contrast, C33A cells expressing MCV LT in the absence of MCV Ori showed a diffuse nuclear RPA70 signal (data not shown). This result suggests that RPA does not localize to a pre-existing sub-nuclear domain, but specifically targets the MCV LT/Ori foci, which are presumably enriched in single-stranded Ori DNA unwound by MCV LT. This suggests that RPA plays a similar role in MCV LT-mediated DNA replication as in SV40 replication. In cells co-expressing the Brd4-LT DNI, RPA70 remained co-localized with MCV LT in the replication foci, suggesting that inhibition of the MCV LT and Brd4 interaction does not affect the ability of MCV LT to unwind viral Ori and recruit RPA70 (Fig. 7B). Instead, the DNI may inhibit recruitment of other replication factor(s) downstream of viral DNA unwinding in the DNA replication pathway.

### Brd4 recruits RFC to MCV viral DNA replication sites

2We further investigated the mechanism by which the Brd4-MCV LT interaction contributes to MCV replication. Brd4 has been shown to interact with RFC, a conserved five-subunit protein complex essential for DNA replication [18]. RFC binds to template-primer junctions and catalyzes the loading of a proliferating cell nuclear antigen (PCNA) clamp onto DNA, thereby recruiting DNA polymerases to the site of DNA synthesis [35], [36]. We therefore tested if the RFC and Brd4 interaction is important for MCV DNA replication.

**Figure 8:**
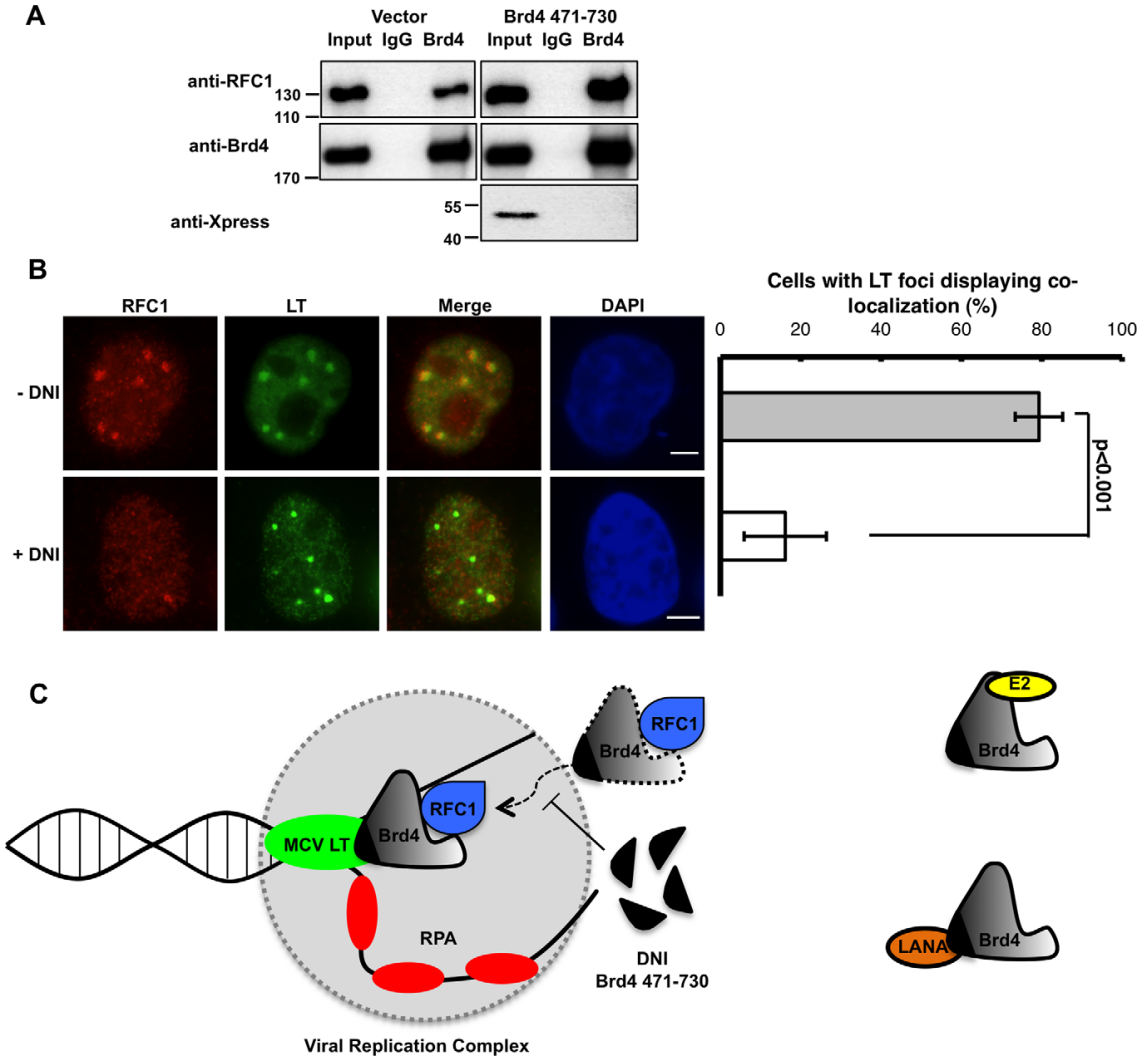
The DNI inhibits RFC1 recruitment to the viral replication complex. **A**. Brd4 interacts with RFC1 and this interaction is not affected by Brd4 471-730. 293T cells were co-transfected with an RFC1 expression plasmid and either a pCDNA4C plasmid encoding Xpress-tagged Brd4 471-730 or the empty vector. Nuclear extracts IPed using either normal rabbit IgG or Brd4 CA were immunoblotted using antibodies against RFC1, Brd4 or the Xpress epitope tag. **B**. The DNI inhibits RFC1 and MCV LT co-localization. C33A cells were co-transfected with pcDNA4C-MCV LT and either pcDNA4C-MCV Ori (−DNI) or pcDNA4C-MCV Ori-DNI (+DNI). Cells were stained using antibodies for RFC1 (Red) and MCV LT (Green). Bar, 5 µm. Mean and standard deviation of the percentage of cells with LT foci displaying RFC1 and LT co-localization in punctate dots were calculated from three independent experiments. More than 100 cells were counted in each sample. **C**. A model for the Brd4-mediated MCV DNA replication. The Brd4-MCV LT interaction recruits RFC1 to the unwinding viral DNA to facilitate replication. The DNI blocks the Brd4 and MCV LT interaction and prevents the recruitment of RFC1 to the viral replication complex. The interactions between Brd4 and other viral proteins such as papillomavirus E2 and KSHV LANA are also shown.

Brd4 interacts with RFC by binding directly to the largest subunit, RFC1 [18]. The RFC1 binding site was mapped to Brd4 aa 350–599 region [18]. This region spans the second bromodomain and a linker upstream of the ET domain (Fig. 3C). The MCV LT binding site in Brd4 was localized to the ET domain as well as the linker (Fig. 3C). Hence, RFC and MCV LT may bind to Brd4 simultaneously, but it is also conceivable that the partial overlap of their binding sites makes this impossible. We therefore tested if RFC could bind to Brd4 471-730, which is directly involved in MCV LT binding. Co-IP showed that RFC1 could bind to Brd4 1-594 fragment, but not to Brd4 471-730 (Fig. S9). Binding of RFC1 to Brd4 1-594 appeared weak, which was also reported in the previous study [18]. We therefore performed Co-IP to test the interaction of endogenous Brd4 with RFC1. Brd4 antibody could efficiently pull down RFC1, suggesting that RFC1 binds much better to full-length Brd4 (Fig. 8A). More importantly, co-expressing Brd4 471-730 did not affect the Brd4 and RFC1 interaction (Fig. 8A). These results demonstrate that Brd4 471-730 does not bind to RFC1 and therefore cannot change its interaction with Brd4. It also indicates that, by blocking the Brd4 and MCV LT interaction, Brd4 471-730 may act as a DNI to prevent the Brd4-mediated recruitment of RFC into the MCV LT/Ori replication complex.

In C33A cells carrying MCV LT and Ori, RFC1 indeed co-localized with MCV LT at replication foci (Fig. 8B, −DNI). Because Brd4 is also recruited to these foci (Fig. 1), this observation is consistent with the idea that RFC1 and MCV LT can interact with Brd4 simultaneously and, through this interaction, Brd4 recruits RFC1 to the MCV LT/Ori replication foci. More importantly, co-expressing the DNI from the MCV Ori vector abolished RFC recruitment to these foci in more than 60% of the cells carrying MCV foci (Fig. 8B). This demonstrates that recruitment of RFC1 to MCV replication foci is dependent upon its interaction with Brd4, and DNI may inhibit MCV replication by blocking recruitment of the Brd4/RFC complex to the MCV Ori replication complex. In summary, this study shows that MCV LT and Brd4 co-localize to the viral Ori foci and may recruit the RFC complex to promote viral DNA replication.

## Discussion

MCV is the first human polyomavirus to be definitively linked to a human cancer [[1] reviewed in [3]]. Although it is well established that the clonal integration of MCV DNA sequences into the host genome precedes development of the majority of MCC cases, the mechanisms of MCV-induced oncogenesis are largely unexplored. Many aspects of the viral life cycle also remain elusive. Elucidation of the life cycle of this newly discovered virus would not only contribute to understanding the oncogenic mechanism of this DNA tumor virus but also identify effective targets for developing therapeutic approaches to cure the oncogenic viral infection.

In SV40, to initiate viral DNA replication, LT assembles as a double hexamer by recognizing the viral Ori through its OBD domain, and its helicase/ATPase domain unwinds the viral DNA, allowing assembly of the cellular DNA-replication machinery [37]. For MCV replication, a 71-bp minimal MCV replication core origin has been identified as the sequence recognized by MCV LT for initiating DNA replication [4]. MCV sT appears to modestly enhance LT-mediated replication of plasmids carrying the MCV Ori. Despite these recent advances, much of the biology of MCV DNA replication has not been mechanistically defined and it is important to note that MCV replicates in cell cultures only at very low levels compared to SV40 [7], [8], [9]. How MCV utilizes the host DNA replication machinery for its own replication remains elusive.

In this study, we showed that the cellular protein Brd4 binds to MCV LT and plays a critical role in MCV DNA replication. The Brd4-MCV LT interaction was confirmed both *in vivo* and *in vitro*. Brd4 and MCV LT colocalize in punctate nuclear foci containing the MCV Ori. Immuno-FISH and BrdU pulse labeling further demonstrated that the Brd4/MCV LT complex is recruited to the MCV DNA replication foci. Brd4 knockdown led to significant inhibition of MCV replication, which could be rescued by recombinant Brd4 *in vitro*. We mapped the MCV LT binding site to the aa 471-730 region of Brd4. Brd4 471-730 functions as a DNI to block the Brd4-LT interaction and to dissociate Brd4 from the MCV LT/Ori foci. Expression of the DNI leads to inhibition of MCV replication both *in vivo* and *in vitro*, abrogating viral genome replication in host cells.

We also applied a chemical compound JQ1(+) to release endogenous Brd4 from chromatin, which led to an enhanced MCV viral DNA replication while not affecting cell division. This result shows that the role of Brd4 in MCV DNA replication could be uncoupled from its function in transcriptional regulation and cell cycle control.

Processive DNA replication in eukaryotes requires highly coordinated actions of many cellular replication factors. Our study sheds light on some key events of MCV replication. In our model (Fig. 8C), MCV LT recognizes the viral replication Ori and functions as a helicase to unwind the viral DNA. RPA is then recruited to replication forks to protect single stranded viral DNA, allowing the assembly of DNA polymerase alpha-primase into the replication complex for synthesizing the primed site. Through direct interactions with MCV LT, Brd4 recruits RFC, which could then catalyze the loading of a PCNA clamp and tethering of DNA polymerase delta for processive DNA elongation. By blocking the Brd4-MCV LT interaction, the DNI prevents recruitment of Brd4-associated RFC into the viral replication complex, causing inhibition of viral DNA replication (Fig. 8C).

The cellular foci detected in cells carrying both MCV LT and Ori do not recruit the host replication helicase MCM2 and yet are positively marked by the ssDNA binding protein RPA 70. Immuno-FISH showed that these foci contain both MCV Ori DNA and MCV LT (Fig. 1D). The presence of RPA 70 suggests that these foci are MCV replication centers with viral DNA Ori unwound and viral replication initiated by the MCV LT helicase, rather than the host DNA replication centers, which would be enriched with MCM2.

RPA 70 has been shown to be essential for SV40 replication *in vitro* [34]. Consistent with this idea, we found that RPA70 is recruited to MCV replication foci, which presumably facilitates viral DNA replication in cells. The DNI expression in cells had no direct effect on RPA 70 localization to the foci but significantly inhibited the assembly of RFC1 into the replication complex. This is consistent with the fact that MCV LT unwinding of the viral DNA and RPA stabilization of ssDNA occur upstream of RFC recruitment.

Brd4 471-730 inhibits the MCV LT and Brd4 interaction in a dose-dependent fashion, and a high concentration of the molecule is needed to achieve complete inhibition (Fig. S5). This observation indicates that Brd4 471-730 may not fold as well as the native fragment present in endogenous full-length Brd4, therefore more molecules are needed to compete with endogenous Brd4 for binding MCV LT. Nonetheless, this molecule provides a useful tool to show that blocking the MCV LT and Brd4 interaction can impair MCV replication. The Brd4 471-730 DNI was able to inhibit more than 50% of the MCV replication (Fig. 5). Consistently, it also inhibited recruitment of both Brd4 and RFC to the MCV LT foci in more than 50% of the foci positive cells (Fig. 4 and 8). These data suggest that blocking the Brd4/RFC and LT interaction may likely be the main mechanism by which the DNI inhibits MCV replication. However, whether DNI binding to MCV LT inhibits an intrinsic replication activity of MCV LT that is independent of its ability to bind Brd4 remains an interesting question to be further investigated in future studies.

The Brd4 interaction with MCV LT can be readily detected by Co-IP and pull down experiments. However, colocalization of Brd4 and MCV LT was clearly detected only when the viral replication Ori were present (Fig. 1C). A similar phenomenon was observed in U2OS cells (data not shown). It is possible that tethering of Brd4 by MCV LT to the MCV replication Ori foci may increase the concentration of this complex at the foci to allow its better detection by IF. Additionally, Brd4's association with acetylated histone is mobile and has been shown to interact with chromatin with a rapid “on and off” mode of binding [38]. The chromatin-bound Brd4 could be readily liberated from chromatin upon signal-triggered histone deacetylation [39]. In the presence of the viral Ori construct, MCV LT is recruited to Ori to initiate viral replication. These molecular events could provide a signal to stimulate dissociation of Brd4 from chromatin, leading to its recruitment to the LT bound replication foci. Together, our observations suggest that MCV LT binding to viral Ori may promote translocation of the Brd4/RFC complex to facilitate viral replication.

Recruitment of Brd4 to MCV LT foci could also be regulated in a cell cycle dependent manner. The MCV LT and viral Ori foci are detected in nearly 30% of the LT positive cells, suggesting that formation of these foci may require the cells to be present in a specific stage. In line with this finding, we observed that cells containing the punctate viral replication foci frequently have enlarged nuclei, indicating ongoing host DNA replication in these cells. Indeed, IF using cell cycle specific makers suggests that MCV LT replication mainly occurs in S phase (data not shown).

This study establishes a novel role of Brd4 in MCV replication. A possible role of Brd4 in papillomavirus replication has been proposed [26], although the underlying mechanism remains to be further investigated. Interestingly, KSHV LANA also binds the Brd4 ET domain [40], suggesting that Brd4 could play a similar role in KSHV replication. The current study therefore provides a paradigm for investigating the role of Brd4 in the replication events of other episomal DNA tumor viruses.

In summary, our study provides mechanistic insight into the machinery of MCV DNA replication. This work also establishes a platform for investigating additional host DNA replication factors that could contribute to MCV replication. Our proof of principle study suggests that blocking the Brd4 and MCV LT interaction could prevent MCV from replicating in host cells (Fig. 5A). MCV is the proven causative agent for MCC and it also globally infects healthy individuals [3]; this study therefore identified the Brd4-MCV LT interaction as an important target for developing effective therapeutic strategies to cure MCV infection and associated cancers.

## Materials and Methods

### Cell lines, transfection, and plasmids

C33A and 293T cells were maintained in Dulbecco's modified Eagle's medium (Invitrogen) containing 10% FBS (Clontech). 293-4T cells stably expressing MCV LT and sT were maintained as described previously [7]. Transfection methods and recombinant plasmid construction details can be found in the Extended Experimental Procedures.

### Immunoprecipitation and GST pull-down

Immunoprecipitation and GST pull-down were carried out as described [11]. Additional experimental details and antibody information are provided under the Extended Experimental Procedures.

### Immunofluorescence and Immuno-FISH

IF was performed as previously described [11]. A complete list of antibodies used is provided under the Extended Experimental Procedures. Immuno-FISH was performed using a published protocol [41]. A specific probe recognizing pcDNA4C-MCV Ori and a control probe recognizing the HPV genome were labeled with Biotin-dUTP (AppliChem) using nick translation. Hybridized probes were detected with the TSA biotin system (PerkinElmer) following the manufacturer's instruction.

### Viral genome re-ligation, quantitative polymerase chain reaction (qPCR) and Southern blotting

To generate the circular MCV genome, pMCV-R17a [42] was digested with EcoRI, diluted to 4 µg/ml and treated with T4 DNA ligase (NEB) at 16°C for 16 h. The DNA was recovered using ethanol precipitation.

For analyzing the MCV genome copies by qPCR, total cellular DNA was extracted by proteinase K digestion, phenol∶chloroform∶isoamylalcohol (25∶24∶1) extraction and ethanol precipitation. qPCR was carried out on a Bio-Rad iQTM 5 multicolor real-time PCR detection system using iQ SYBR Green Supermix (Bio-Rad) following the manufacturer's instructions. 500 ng total DNA was added as template in each reaction. The primers amplifying the MCV genome have been described previously [7]. The MCV qPCR values were normalized to the beta-actin result. All experiments were performed in triplicates and repeated three times. The data were analyzed using Bio-Rad iQ5 software.

Southern blotting was performed as described previously with minor modification [43]. 10 µg total DNA was digested with EcoR I, treated with or without DpnI at 37°C for 2 h, and separated on a 0.7% agarose gel. Probes recognizing the MCV genome in pMCV-R17a were labeled with [α-^32^P] dCTP (3000 Ci/mmol) using Prime-It II random primer labeling kit (Agilent Technologies) per the manufacturer's instructions. The results were analyzed using a Phosphorimager (Typhoon 9400; GE Healthcare).

### *In vitro* replication

*In vitro* replication was performed as described previously with minor modification [29]. 293T cell extracts were prepared 48 h after transfection with pcDNA4C-MCV LT, encoding full-length MCV LT with silent mutations that prevent splicing of the 57 kT intron [7]. Standard reaction mixture (50 µl) contained 40 mM creatine phosphate (pH 7.7, di-Tris salt); 7 mM MgCl_2_; 0.5 mM DTT; 4 mM ATP; 200 µM each CTP, UTP, GTP; 80 µM each dATP, dTTP, dGTP; 3.3 µM [α-^32^P] dCTP (3000 Ci/mmol); 100 µg/ml creatine kinase; 300 ng pMCV-R17a and 280–300 µg total cell extracts. For SV40-Ori replication, cell extracts were prepared from 293T without transfection, and 300 ng pEGFP-C1 was added as template. The DNA digested with XhoI was separated on a 1% agarose gel and detected using autoradiography. Brd4 471-730 protein used for the *in vitro* replication was expressed in *E.coli* and purified using glutathione agarose. Full-length Brd4 used in the rescue experiment was expressed in Sf9 insect cells.

### FACS, image analysis and statistical analysis

To perform the fluorescence-activated cell sorting, cells were fixed with 70% ethanol and stained with 25 µg/ml propidium iodide. RNA was digested with 100 µg/ml RNase A. FACS results were analyzed using FlowJo software.

Cells from IF were examined using an Olympus IX81 inverted fluorescence microscope and associated Slidebook 5.0 software. Cell images were cropped using ImageJ. Intensities of Western blot and autoradiography signal were analyzed using ImageJ.

Each of the qPCR and cell counting experiments were performed in triplicates. More than 100 cells were counted in each sample. Mean and standard deviation shown in the figures were calculated from three independent experiments. Two-tailed t-tests were performed and the P-values are shown in the figures.

## Supporting Information

Figure S1 **Purification of Brd4 functional complex using a proteomic approach.** The human Brd4 gene was subcloned in-frame with a highly specific Xpress tag. 293T cells were transfected with the Xpress-Brd4 construct (Brd4) or an empty vector (V). Brd4 and associated proteins were affinity purified from nuclear lysates using Xpress antibody-conjugated sepharose. Proteins co-purified with Xpress-Brd4 were analyzed on SDS-PAGE and identified by mass spectrometry. This mass spectrometry identified four peptides that match the SV40 LT protein.
(PDF)

Figure S2 **Interaction of Brd4 and MCV LT.** **A**. MCV LT specifically interacts with human Brd4. 293T cells were transfected with pMtB (an sT expression plasmid, sT) or pADL* (LT). Nuclear extracts IPed with normal rabbit IgG (IgG) or Brd4NA antibody (Brd4) were immunoblotted using anti-Brd4 antibody or mouse monoclonal antibody 2t2, which is specific for the common leader peptide present on both sT and LT. Plasmid pMtB expresses a doublet sT band, which represents full length MCV sT protein (22 kD) together with a slower-migrating band (24 kD) that appears to be expressed from a spliced mRNA in which the LT splice donor is spliced in frame with the blasticidin resistance ORF (unpublished results). A very small amount of the sT doublet and a proteolytic cleavage product of MCV LT carrying the N-terminal region recognized by the 2t2 antibody was detected in Brd4 IP, supporting that the N-terminal half of MCV LT is involved in binding Brd4, as observed in Fig. 3. **B**. GST-LT pull-down Brd4. 293T cells were transfected with pcDNA4C-Brd4. Nuclear extracts were mixed with either immobilized bacterially-produced GST or GST-LT. The pull-down samples and nuclear extracts (input) were immunoblotted using Brd4NA. GST or GST-LT eluted from the glutathione resin was separated by SDS-PAGE and stained with Coomassie Brilliant Blue (CBB). Also see Fig. 3. **C**. MCV-LT and Brd4 affinity analysis. 293T cells were transfected with pcDNA4C-MCV LT. Brd4 and MCV LT Co-IP was performed as in **A**. The Brd4-MCV LT immuno-complexes were incubated with buffer containing increasing salt concentrations. 20 µg nuclear protein input, 50% of protein washed off the beads (WA) and 20% of proteins retained on the beads were immunoblotted for MCV LT and Brd4.
(PDF)

Figure S3 **Brd4 knockdown inhibits MCV DNA replication and the inhibition can be rescued** ***in vitro*** **by recombinant Brd4.** **A**. C33A cells were transfected with a Brd4 siRNA (K.D.) or a control siRNA (C.O.). At 30 h p.t., cells were re-transfected with pcDNA4C-MCV LT and pcDNA4C-MCV Ori. At 78 h p.t., cells were double-stained with Brd4 CA (Red) and MCV LT antibody (Green). Cells were also counter-stained with DAPI. Bar, 5 µm. **B**. 293T cells were transfected with a Brd4 siRNA or a control siRNA. At 40 h p.t., cells were re-transfected with pcDNA4C-MCV LT. Cell extracts were prepared at 88 h p.t. and used for *in vitro* replication of MCV DNA as shown in Fig. 2C–E. Cell extracts used in the replication assay were immunoblotted for Brd4, MCV LT and GAPDH. Shown are the representative immunoblots of several repeated experiments. **C**. His-Brd4 purified from insect cells using nickel resin and an equal amount of nonspecific proteins eluted from the nickel resin incubated with insect cells carrying wild-type baculovirus was analyzed using anti-Brd4 immunoblotting (control: wild type baculovirus, rBrd4: baculovirus expressing Brd4). Asterisk marks the position of a rBrd4 proteolytic cleavage product. **D**. Dose-dependent rescue of *in vitro* viral replication by the recombinant Brd4 protein. Extracts from cells transfected with a Brd4 siRNA and pcDNA4C-MCV LT as described in **B** were used in the *in vitro* replication assay. Increasing amounts of recombinant His-Brd4 was added to the reactions. All reactions were performed in triplicates. **E**. Analysis of purified Brd4 protein used in Fig. 2D and 2E. To purify Brd4 for the rescue experiment, 293T cells were transfected with a plasmid IIT-Brd4, which encodes Brd4 fused to two IgG binding domains of protein A through a tobacco etch virus (TEV) protease cleavage site, or an empty vector. At 48 h p.t., IIT-Brd4 was purified using IgG sepharose and released by TEV protease digestion. Purified Brd4 and an equal amount of nonspecific proteins isolated from the vector control cells were analyzed in Coomassie Brilliant Blue staining.
(PDF)

Figure S4 **GST-Brd4 471-730 pulls down MCV LT** ***in vitro*** **.** GST and GST tagged Brd4 471-730 were expressed in *E.coli* and immobilized on glutathione beads. GST was used as a negative control. The purified proteins were analyzed on SDS-PAGE and stained with Coomassie Brilliant Blue. MCV LT was translated and labeled with ^35^S-Met using *in vitro* transcription and translation, and tested for binding to the immobilized GST fusion proteins. The pull-down and input samples were resolved on SDS-PAGE and LT was detected by autoradiography.
(PDF)

Figure S5 **Brd4 471-730 inhibits the MCV LT and Brd4 interaction in a dose-dependent manner.** 293T cells were co-transfected with pcDNA4C-MCV LT and increasing amount of pcDNA4C-Brd4 471-730. pcDNA4C vector was added to reach the same amount of total plasmids used in each transfection. Nuclear extracts IPed using either normal rabbit IgG or Brd4 NA were immunoblotted using an antibody against MCV LT, Brd4, actin or the Xpress epitope tag carried by the Brd4 truncation mutant.
(PDF)

Figure S6 **DNI expression from pcDNA4C-MCV Ori-DNI construct in C33A cells.** **A**. Cells were transfected with either pcDNA4C-MCV Ori (Origin) or pcDNA4C-MCV Ori-DNI (Origin-DNI). Non-transfected cells were used as control. 10 µg total proteins were loaded in each lane and immunoblotted for Xpress tagged DNI, and actin. **B**. Cells were transfected with pcDNA4C-MCV Ori-DNI (DNI). At 48 h p.t., cells were stained with anti-Xpress antibody (green) and DAPI (blue). Bar, 5 µm.
(PDF)

Figure S7 **MCV and host DNA replication detected by BrdU labeling.** **A**. Kinetics of MCV DNA replication detected by BrdU pulse labeling. C33A cells were transfected with pcDNA4C-MCV LT, pEGFP-MCV LT and either pcDNA4C-MCV Ori (without DNI) or pcDNA4C-MCV Ori-DNI (with DNI). GFP-MCV LT was used to mark the replication foci. At 44 h p. t., cells were pulsed with 10 µM BrdU for 0, 2, 5, 10, 15, or 20 minutes and fixed with acetone. Cells were stained with anti-BrdU antibodies. Mean and standard deviation of the percentage of cells with LT foci displaying BrdU in punctate dots were calculated from three independent experiments. More than 100 cells were counted in each sample. **B**. The DNI does not interfere with cellular DNA replication. C33A cells were transfected as indicated using plasmids: pcDNA4C-MCV Ori-DNI (Ori-DNI), pADL* (LT), pcDNA4C-MCV Ori (Ori), pcDNA4C-DNI (DNI) or pcDNA4C (Vector). At 36 h p. t., cells were pulsed with 10 µM BrdU for 30 min and fixed with 3% PFA. DNA was denatured with 2 M HCl on ice for 10 min before BrdU staining. Mean and standard deviation of the percentage of BrdU positive cells were calculated from three independent experiments. Anti-Xpress IF staining was also performed on these cells to show that the DNI transfection efficiency is ∼40% for all samples.
(PDF)

Figure S8 **Brd4 471-730 ameliorates the cytotoxicity of MCV LT.** 293T cells were co-transfected with either pOZN (grey) or pOZN-MCV LT (white) together with pcDNA4C (Vector) or pcDNA4C-Brd4 471-730. Cells were counted at 48 h p.t. and normalized to the number obtained from cells co-transfected with pOZN and pcDNA4C.
(PDF)

Figure S9 **Brd4 interacts with RFC1 but this interaction is not mediated by the DNI region of Brd4.** 293T cells were co-transfected with an RFC1 expression plasmid and either a pOZN construct encoding a FLAG-HA tagged Brd4 truncation mutant or the FLAG-HA vector alone. Nuclear extracts IPed with anti-FLAG beads were immunoblotted with HA and RFC1 antibodies.
(PDF)

Text S1 **This section provides a more detailed description of the protocols, experimental procedures and specifics of how the DNA constructs were generated.** It also includes information on materials used in the study and references.
(PDF)
