# Supplementary material for: Bromodomain Protein Brd4 Plays a Key Role in Merkel Cell Polyomavirus DNA Replication
Source: PLoS Pathog. 2012 Nov 8;8(11):e1003021. doi: 10.1371/journal.ppat.1003021 (PMC3493480; doi:10.1371/journal.ppat.1003021)
Supplement: Figure S1 — Purification of Brd4 functional complex using a proteomic approach. The human Brd4 gene was subcloned in-frame with a highly specific Xpress tag. 293T cells were transfected with the Xpress-Brd4 construct (Brd4) or an empty vector (V). Brd4 and associated proteins were affinity purified from nuclear lysates using Xpress antibody-conjugated sepharose. Proteins co-purified with Xpress-Brd4 were analyzed on SDS-PAGE and identified by mass spectrometry. This mass spectrometry identified four peptides that match the SV40 LT protein. (PDF) [file ppat.1003021.s001.pdf]

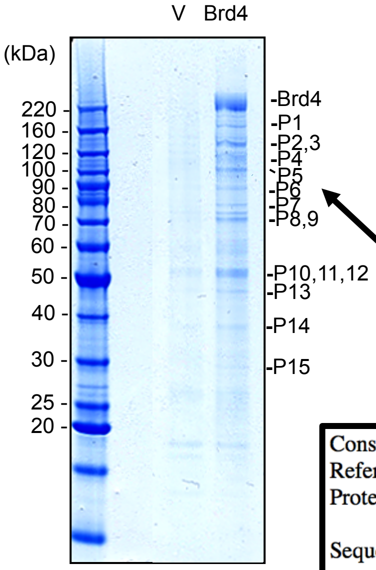

Consensus Group = Z
Reference ID = gil109727197
Protein Name = **large T antigen [Simian virus 40]**

Sequence	Accession	Scan	Charge	Sf	MH+	Xcorr	deltaCn	Sp	TIC
ALNVNLPLDR	gil109727197	2781	2	0.83	1124.6	3.13	0.18	1056	38958
EYLMYSALTR	gil109727197	2950	2	0.55	1247.1	2.70	0.20	394	11428
TQIFPPGIVTM*NEYSVPK	gil109727197	3091	2	0.53	2037.9	3.34	0.27	315	4607
TTLAAALLELCGGK	gil109727197	3555	2	0.38	1416.7	3.04	----	654	6515
