# Supplementary figures and images for: Bromodomain Protein Brd4 Plays a Key Role in Merkel Cell Polyomavirus DNA Replication
Source: PLoS Pathog. 2012 Nov 8;8(11):e1003021. doi: 10.1371/journal.ppat.1003021 (PMC3493480; doi:10.1371/journal.ppat.1003021)

**A**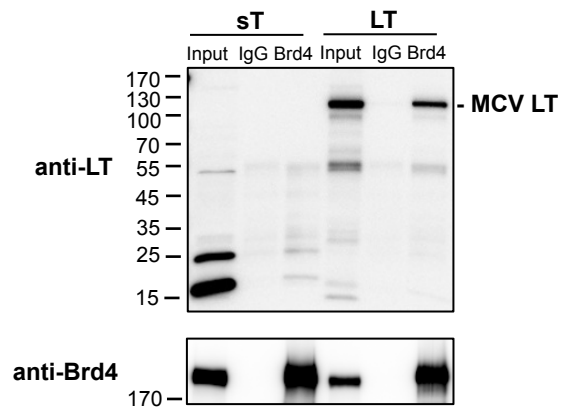**B**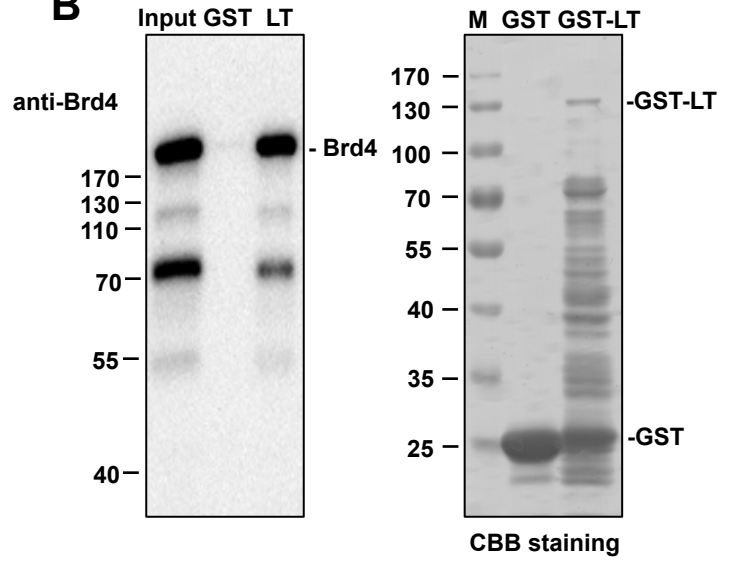**C**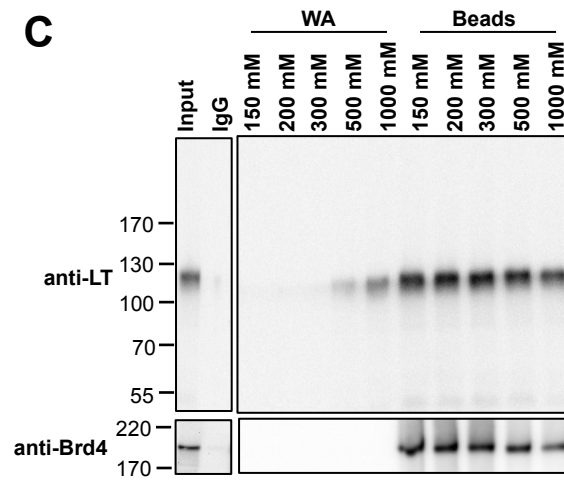

Supplement: Figure S2 — Interaction of Brd4 and MCV LT. A. MCV LT specifically interacts with human Brd4. 293T cells were transfected with pMtB (an sT expression plasmid, sT) or pADL* (LT). Nuclear extracts IPed with normal rabbit IgG (IgG) or Brd4NA antibody (Brd4) were immunoblotted using anti-Brd4 antibody or mouse monoclonal antibody 2t2, which is specific for the common leader peptide present on both sT and LT. Plasmid pMtB expresses a doublet sT band, which represents full length MCV sT protein (22 kD) together with a slower-migrating band (24 kD) that appears to be expressed from a spliced mRNA in which the LT splice donor is spliced in frame with the blasticidin resistance ORF (unpublished results). A very small amount of the sT doublet and a proteolytic cleavage product of MCV LT carrying the N-terminal region recognized by the 2t2 antibody was detected in Brd4 IP, supporting that the N-terminal half of MCV LT is involved in binding Brd4, as observed in Fig. 3. B. GST-LT pull-down Brd4. 293T cells were transfected with pcDNA4C-Brd4. Nuclear extracts were mixed with either immobilized bacterially-produced GST or GST-LT. The pull-down samples and nuclear extracts (input) were immunoblotted using Brd4NA. GST or GST-LT eluted from the glutathione resin was separated by SDS-PAGE and stained with Coomassie Brilliant Blue (CBB). Also see Fig. 3. C. MCV-LT and Brd4 affinity analysis. 293T cells were transfected with pcDNA4C-MCV LT. Brd4 and MCV LT Co-IP was performed as in A. The Brd4-MCV LT immuno-complexes were incubated with buffer containing increasing salt concentrations. 20 µg nuclear protein input, 50% of protein washed off the beads (WA) and 20% of proteins retained on the beads were immunoblotted for MCV LT and Brd4. (PDF) [file ppat.1003021.s002.pdf]

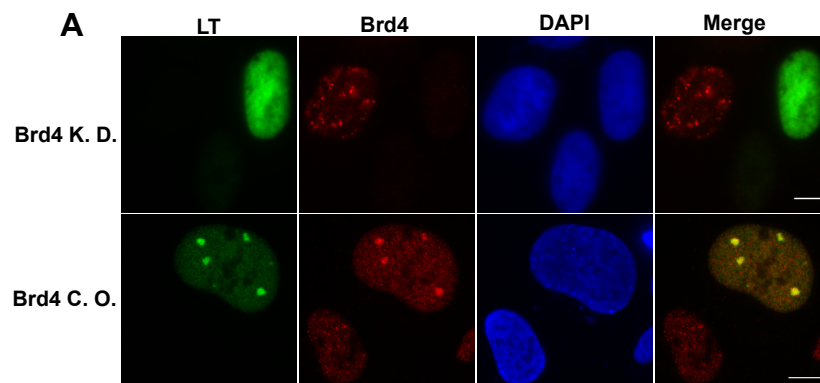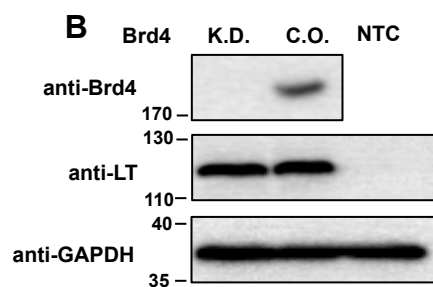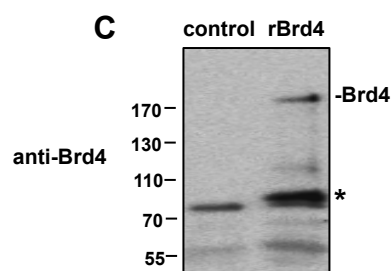

#### D Dose-dependent rescue with rBrd4

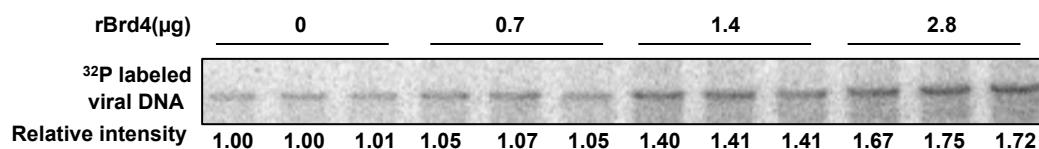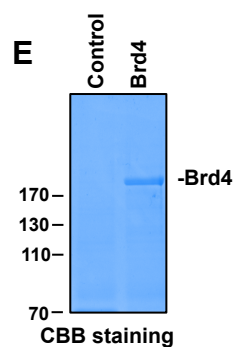

Supplement: Figure S3 — Brd4 knockdown inhibits MCV DNA replication and the inhibition can be rescued in vitro by recombinant Brd4. A. C33A cells were transfected with a Brd4 siRNA (K.D.) or a control siRNA (C.O.). At 30 h p.t., cells were re-transfected with pcDNA4C-MCV LT and pcDNA4C-MCV Ori. At 78 h p.t., cells were double-stained with Brd4 CA (Red) and MCV LT antibody (Green). Cells were also counter-stained with DAPI. Bar, 5 µm. B. 293T cells were transfected with a Brd4 siRNA or a control siRNA. At 40 h p.t., cells were re-transfected with pcDNA4C-MCV LT. Cell extracts were prepared at 88 h p.t. and used for in vitro replication of MCV DNA as shown in Fig. 2C–E. Cell extracts used in the replication assay were immunoblotted for Brd4, MCV LT and GAPDH. Shown are the representative immunoblots of several repeated experiments. C. His-Brd4 purified from insect cells using nickel resin and an equal amount of nonspecific proteins eluted from the nickel resin incubated with insect cells carrying wild-type baculovirus was analyzed using anti-Brd4 immunoblotting (control: wild type baculovirus, rBrd4: baculovirus expressing Brd4). Asterisk marks the position of a rBrd4 proteolytic cleavage product. D. Dose-dependent rescue of in vitro viral replication by the recombinant Brd4 protein. Extracts from cells transfected with a Brd4 siRNA and pcDNA4C-MCV LT as described in B were used in the in vitro replication assay. Increasing amounts of recombinant His-Brd4 was added to the reactions. All reactions were performed in triplicates. E. Analysis of purified Brd4 protein used in Fig. 2D and 2E. To purify Brd4 for the rescue experiment, 293T cells were transfected with a plasmid IIT-Brd4, which encodes Brd4 fused to two IgG binding domains of protein A through a tobacco etch virus (TEV) protease cleavage site, or an empty vector. At 48 h p.t., IIT-Brd4 was purified using IgG sepharose and released by TEV protease digestion. Purified Brd4 and an equal amount of nonspecific proteins isolated from the v [file ppat.1003021.s003.pdf]

CBB staining

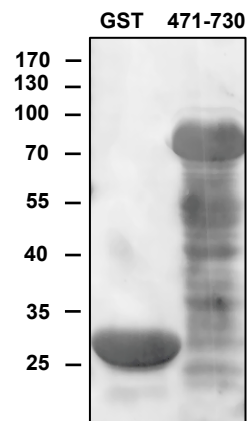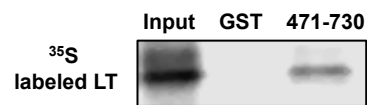

Supplement: Figure S4 — GST-Brd4 471-730 pulls down MCV LT in vitro . GST and GST tagged Brd4 471-730 were expressed in E.coli and immobilized on glutathione beads. GST was used as a negative control. The purified proteins were analyzed on SDS-PAGE and stained with Coomassie Brilliant Blue. MCV LT was translated and labeled with 35S-Met using in vitro transcription and translation, and tested for binding to the immobilized GST fusion proteins. The pull-down and input samples were resolved on SDS-PAGE and LT was detected by autoradiography. (PDF) [file ppat.1003021.s004.pdf]

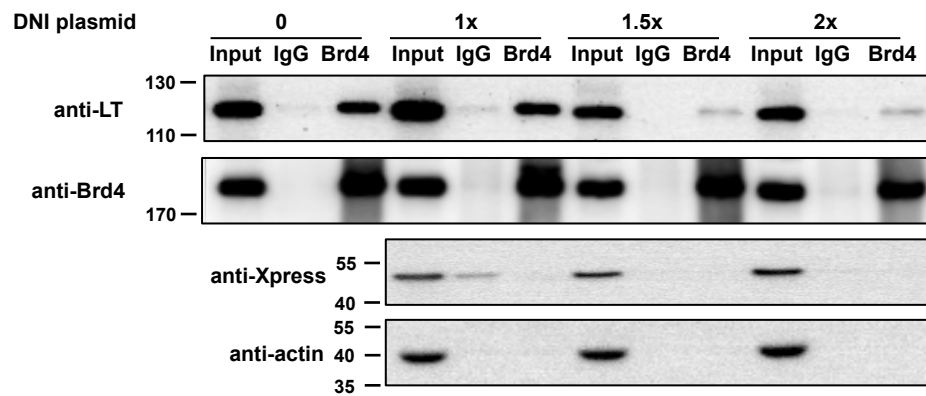

Supplement: Figure S5 — Brd4 471-730 inhibits the MCV LT and Brd4 interaction in a dose-dependent manner. 293T cells were co-transfected with pcDNA4C-MCV LT and increasing amount of pcDNA4C-Brd4 471-730. pcDNA4C vector was added to reach the same amount of total plasmids used in each transfection. Nuclear extracts IPed using either normal rabbit IgG or Brd4 NA were immunoblotted using an antibody against MCV LT, Brd4, actin or the Xpress epitope tag carried by the Brd4 truncation mutant. (PDF) [file ppat.1003021.s005.pdf]

**A**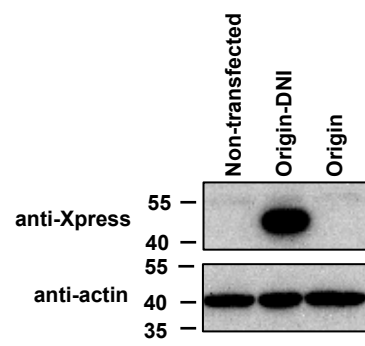**B**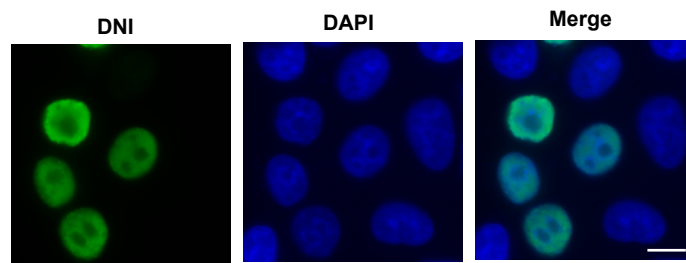

Supplement: Figure S6 — DNI expression from pcDNA4C-MCV Ori-DNI construct in C33A cells. A. Cells were transfected with either pcDNA4C-MCV Ori (Origin) or pcDNA4C-MCV Ori-DNI (Origin-DNI). Non-transfected cells were used as control. 10 µg total proteins were loaded in each lane and immunoblotted for Xpress tagged DNI, and actin. B. Cells were transfected with pcDNA4C-MCV Ori-DNI (DNI). At 48 h p.t., cells were stained with anti-Xpress antibody (green) and DAPI (blue). Bar, 5 µm. (PDF) [file ppat.1003021.s006.pdf]

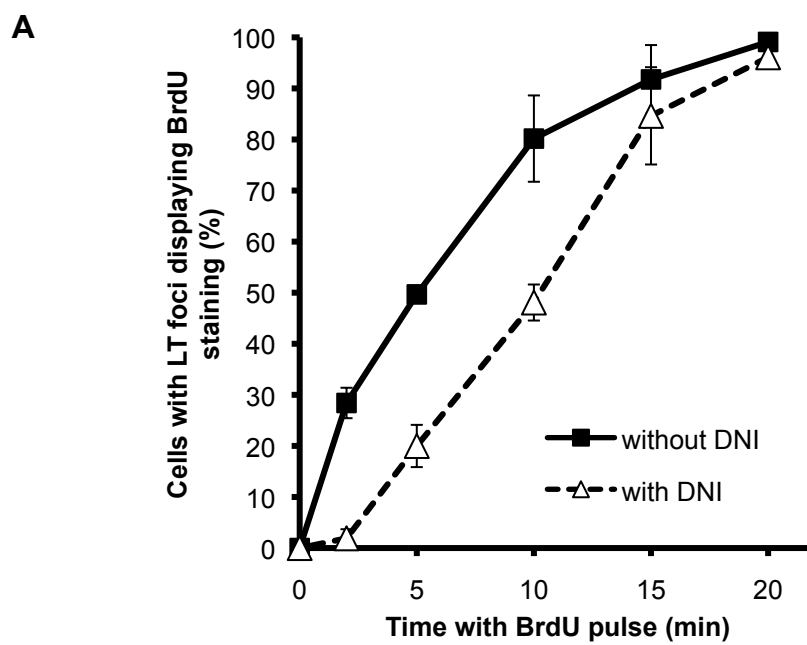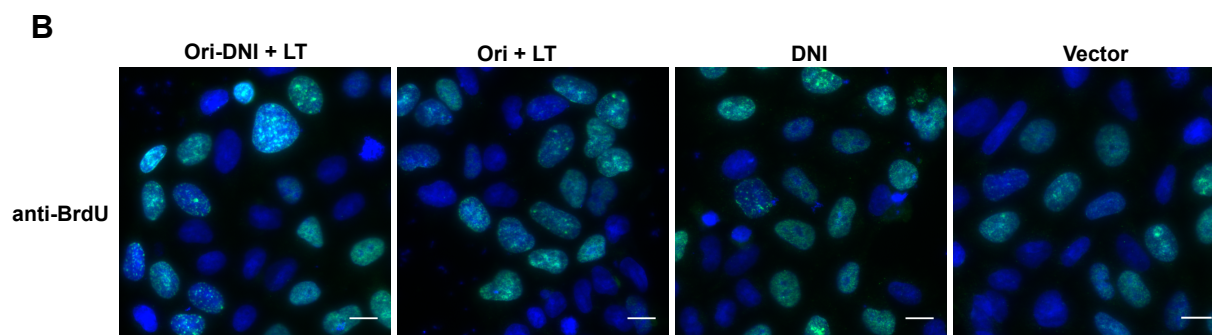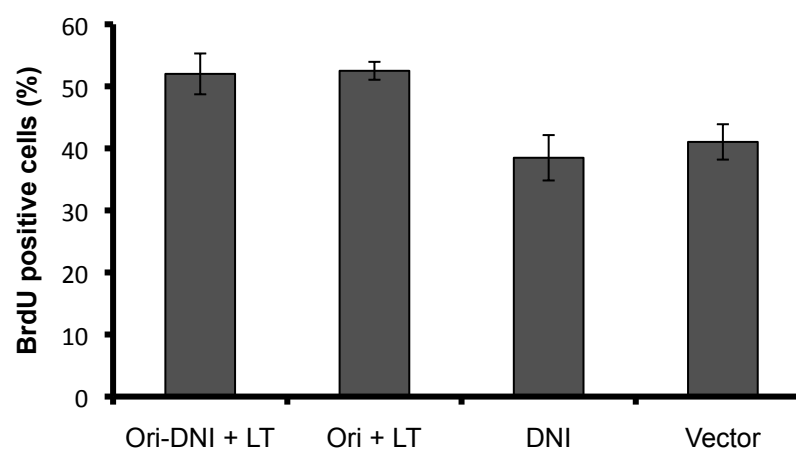

Supplement: Figure S7 — MCV and host DNA replication detected by BrdU labeling. A. Kinetics of MCV DNA replication detected by BrdU pulse labeling. C33A cells were transfected with pcDNA4C-MCV LT, pEGFP-MCV LT and either pcDNA4C-MCV Ori (without DNI) or pcDNA4C-MCV Ori-DNI (with DNI). GFP-MCV LT was used to mark the replication foci. At 44 h p. t., cells were pulsed with 10 µM BrdU for 0, 2, 5, 10, 15, or 20 minutes and fixed with acetone. Cells were stained with anti-BrdU antibodies. Mean and standard deviation of the percentage of cells with LT foci displaying BrdU in punctate dots were calculated from three independent experiments. More than 100 cells were counted in each sample. B. The DNI does not interfere with cellular DNA replication. C33A cells were transfected as indicated using plasmids: pcDNA4C-MCV Ori-DNI (Ori-DNI), pADL* (LT), pcDNA4C-MCV Ori (Ori), pcDNA4C-DNI (DNI) or pcDNA4C (Vector). At 36 h p. t., cells were pulsed with 10 µM BrdU for 30 min and fixed with 3% PFA. DNA was denatured with 2 M HCl on ice for 10 min before BrdU staining. Mean and standard deviation of the percentage of BrdU positive cells were calculated from three independent experiments. Anti-Xpress IF staining was also performed on these cells to show that the DNI transfection efficiency is ∼40% for all samples. (PDF) [file ppat.1003021.s007.pdf]

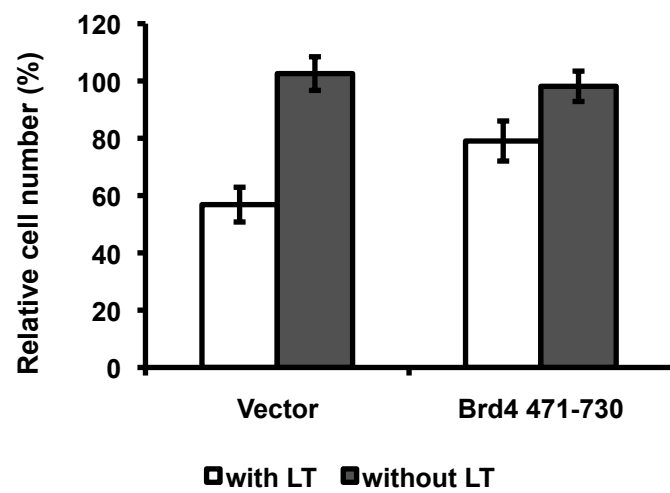

Supplement: Figure S8 — Brd4 471-730 ameliorates the cytotoxicity of MCV LT. 293T cells were co-transfected with either pOZN (grey) or pOZN-MCV LT (white) together with pcDNA4C (Vector) or pcDNA4C-Brd4 471-730. Cells were counted at 48 h p.t. and normalized to the number obtained from cells co-transfected with pOZN and pcDNA4C. (PDF) [file ppat.1003021.s008.pdf]

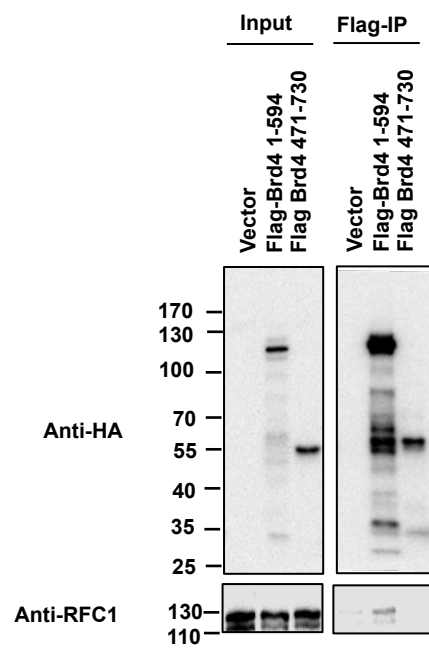

Supplement: Figure S9 — Brd4 interacts with RFC1 but this interaction is not mediated by the DNI region of Brd4. 293T cells were co-transfected with an RFC1 expression plasmid and either a pOZN construct encoding a FLAG-HA tagged Brd4 truncation mutant or the FLAG-HA vector alone. Nuclear extracts IPed with anti-FLAG beads were immunoblotted with HA and RFC1 antibodies. (PDF) [file ppat.1003021.s009.pdf]
