# Supplementary material for: Bromodomain Protein Brd4 Plays a Key Role in Merkel Cell Polyomavirus DNA Replication
Source: PLoS Pathog. 2012 Nov 8;8(11):e1003021. doi: 10.1371/journal.ppat.1003021 (PMC3493480; doi:10.1371/journal.ppat.1003021)
Supplement: Text S1 — This section provides a more detailed description of the protocols, experimental procedures and specifics of how the DNA constructs were generated. It also includes information on materials used in the study and references. (PDF) [file ppat.1003021.s010.pdf]

Supplemental Information

Extended Experimental Procedures

This section provides a more detailed description of the protocols and experimental procedures used in this study. It also includes specifics of how the DNA constructs were generated. Additional information on antibodies, siRNAs and other materials used in the study are also included.

Cell culture, siRNA/DNA transfection and JQ1 treatment

C33A and 293T cells were maintained in Dulbecco's modified Eagle's medium (Invitrogen) containing 10% FBS (Clonetech). 293-4T cells stably expressing MCV LT and sT were maintained as described previously [1]. Sf9 insect cells were maintained in Sf-900 II SFM (Invitrogen) at 27°C.

For IF staining, C33A cells were transfected at 40-50% confluency using Fugene 6 (Roche) and fixed at 48~72 h p.t. For analyzing the viral episome copy numbers, 293-4T cells were transfected using Lipofectamine 2000 (Invitrogen). Cells were harvested at the time points indicated in Fig. 5A. For IP analysis, 293T cells were transfected at 40-50% confluency using the calcium phosphate method as described previously [2]. Cells were harvested at 48 h p.t. For siRNA transfection, 293T cells were transfected at about 40% confluency using the calcium phosphate method [2], and C33A cells were transfected using DhamaFECT II siRNA transfection reagent (Thermo Scientific Dharmacon). To obtain recombinant baculovirus, Sf9 cells were transfected using Cellfectin II (Invitrogen). Unless otherwise indicated, all transfection reactions were performed following the manufacturers' instructions.

JQ1(+) and JQ1(-) were kindly provided by Dr. James E. Bradner (Dana-Farber Cancer Institute). The compounds were dissolved in DMSO. Cells were treated with 300 nM JQ 1(+), JQ1(-) or equal volume of DMSO in culture medium for 24 h.

To label the replicating DNA, at 44 h p.t., cells were pulsed with 10 μM BrdU dissolved in DMSO for 20 min, and cultured with normal growth medium for another hour. Cells were fixed with acetone for 5 min at -20°C before staining with anti-BrdU antibody.

Antibodies and siRNAs

Antibodies used in this study are: anti-actin (Chemicon), anti-HA-HRP (Roche), anti-Xpress (Invitrogen), anti-MCV LT (CM2B4) (Santa Cruz), anti-RFC1 (H-300) (Santa Cruz), anti-RPA70 (Cell signaling), anti-BrdU (Invitrogen) and anti-MCM2 (Cell signaling). The anti-Brd4 NA and anti-Brd4 CA [2] are rabbit polyclonal antibodies specific for Brd4 aa 156-284 and aa 1313-1362, respectively. Monoclonal antibody 2t2 was raised against a maltose binding protein/sT recombinant protein antigen [1]. Splenocytes from vaccinated mice were fused to Sp2/mIL-6 cells (ATCC) and grown with Hybridoma Fusion and Cloning Supplement (Roche) according to manufacturer's instructions. The mAb was found to be specific for the common J-domain leader peptide found on both sT and LT proteins. siGENOME siRNA (D-004937-02-0020) targeting human *BRD4* and control no-targeting siRNA#1 were purchased from Thermo Scientific Dharmacon.

Recombinant plasmid construction

The RFC1 expression construct was obtained from openbiosystems (cat. no.: MHS1010-9205277). The MCV LT expression plasmid pMtB, pADL* and the MCV genomic clone pMCV-R17a have been described previously [1,3]. The previously-reported plasmid pMtB encodes MCV small T-antigen [1]. The plasmid pT+Ori was constructed by transferring the MCV NCCR and early region of MCV isolate R17b into a derivative of pHyg-EGFP (Clontech). The LT gene in pT+Ori contains the same silent modifications found in pADL* that prevent splicing of the 57kT intron. Maps of each of these plasmids can be found at the website <http://home.ccr.cancer.gov/Lco/support.htm>. The cDNA for full-length human Brd4 and sub-fragments were amplified by PCR and cloned into pcDNA4/HisMax C vector (pcDNA4C, Invitrogen) using BamHI and Not I digestion. Proteins expressed from these constructs carry an Xpress tag on the N-terminus, which can be detected by anti-Xpress antibody in both IF and Western blotting. pGex-6P-1-Brd4 595-730 and pGex-6P-1-Brd4 471-730 encoding GST tagged Brd4 fragments have been described previously [4]. To generate pcDNA4C-MCV LT, pOZN-MCV LT and pGEX-MCV LT, the DNA coding

sequence for full-length MCV LT was PCR amplified from pADL*, and inserted into the KpnI/XhoI sites of pcDNA4C or the NotI/XhoI sites of pOZN and pGEX-6P-1 (GE Healthcare), respectively. Protein expression from the pcDNA4C construct is controlled by both a CMV promoter and a T7 promoter, so pcDNA4C-MCV LT was used for *in vitro* transcription and translation. The 97bp replication Origin (5356 nt-65 nt) of MCV [5] was PCR amplified from pMCV-R17a and inserted into the EcoRI site of pcDNA4C to obtain pcDNA4C-MCV Ori. The Brd4 471-730 expression cassette was cut from pcDNA4C using MfeI/XhoI digestion and inserted into pcDNA4C-MCV Ori to generate pcDNA4C-MCV Ori-DNI. To express full-length human Brd4 in Sf9 cells, the Brd4 expression cassette was cut from pcDNA4C-Brd4FL using MfeI/XhoI and subcloned into the EcoRI/XhoI sites of pFastBac1 (Invitrogen), which is used as the donor plasmid in the Bac-to-Bac baculovirus expression system. The recombinant bacmid AcBrd4FL was produced and purified using PureLink HiPure plasmid miniprep kit following the manufacturer's instruction (Invitrogen). All plasmid constructs were verified by DNA sequencing.

Protein expression and purification

GST-MCV LT and GST-Brd4 471-730 fusion proteins were produced in *E. coli* using the respective pGEX plasmids. Cell pellets were lysed in L buffer (50 mM Tris, 0.1 mM EDTA, 2 mM dithiothreitol [DTT], 0.4 mg/ml lysozyme, 0.2 mM phenylmethylsulfonyl fluoride [PMSF], 10 mM magnesium chloride ($MgCl_2$) and 20ug/ml DNase I, pH 7.6) supplemented with protease inhibitors (Roche). GST fusion proteins were purified from the cell lysate using glutathione agarose (Sigma) according to the manufacturer's instructions. Brd4 471-730 was released from glutathione agarose using PreScission protease (GE lifesciences).

Full-length Brd4 was expressed in Sf9 insect cells. Recombinant virus AcBrd4 for expressing His tagged full-length human Brd4 and the empty control virus were generated using the Bac-to-Bac baculovirus expression system (Invitrogen). Full-length Brd4 protein was expressed as described previously [6]. The protein was purified using magnetic nickel beads (Millipore) following the manufacturer's protocols. Purified proteins were dialyzed in D buffer (20 mM HEPES, 150 mM potassium chloride [KCl],

pH 8.0) at 4°C for 3 hours. Expressed proteins were analyzed using sodium dodecyl sulfate polyacrylamide gel electrophoresis (SDS-PAGE), Coomassie Brilliant blue staining and/or Western blotting.

Brd4 was expressed in 293T cells using pLPCX-IIT-Brd4, in which Brd4 is fused to two IgG binding domains of Staphylococcus aureus protein A through a tobacco etch virus (TEV) protease cleavage site. Nucleus were isolated after cell were incubated with buffer A (10 mM HEPES, 10 mM KCl, 0.1 mM EDTA, 1.8% NP40 and 0.2 mM PMSF, pH 7.9, supplemented with protease inhibitors). Nuclear proteins were extracted using buffer B (20 mM HEPES, 400 mM NaCl and 1 mM EDTA, pH 7.9, supplemented with protease inhibitors). After centrifugation at 14000 rpm for 10min at 4°C, the supernatant was diluted 2.7-fold with buffer A. Tagged Brd4 was purified with IgG beads. After the beads were washed three times with 0.15 M KCl Base Buffer (20 mM Tris, 150 mM KCl, 5 mM $MgCl_2$, 0.2 mM PMSF, 10% glycerol, 0.1% Tween-20, pH 8.0, supplemented with protease inhibitors) and twice with 0.4 M KCl Buffer (20mM Tris, 400 mM KCl, 5 mM $MgCl_2$, 0.2 mM PMSF, 10% glycerol, 0.1% Tween-20, pH 8.0, supplemented with protease inhibitors), Brd4 was released from IgG beads using TEV digestion following the manufacturer's instruction. The empty vector pLPCX-IIT was used as a control to transfect 293T cells.

Immunoprecipitation and GST pull-down

Cells harvested at 48 h p.t. were re-suspended in buffer A (10 mM HEPES, 10 mM KCl, 0.1 mM EDTA, 0.1 mM EGTA, 0.2 mM PMSF and 1 mM DTT, supplemented with protease inhibitors, pH 7.9), incubated on ice for 10 min, vortexed 10 s after adding 0.6% NP-40 and centrifuged at 5,000 rpm for 5 min at 4°C. The nuclear pellets were re-suspended in ice-cold buffer B (10 mM HEPES, 200 mM sodium chloride [NaCl], 3 mM $MgCl_2$, 0.2 mM PMSF and 1 mM DTT, pH 7.9, supplemented with protease inhibitors), passed through a 22-gauge needle ten times and extracted at 4°C for 1 h with rotation. After centrifugation at 14,000 rpm for 15 min, soluble proteins were diluted in buffer A to a final salt concentration of 150 mM. Samples were pre-cleared with 3 µg normal rabbit IgG and 10 µl nProtein A Sepharose 4 Fast Flow (GE Healthcare) at 4°C for 1 h with rotation. Supernatants were mixed with 2 µg antibody and rotated at 4°C for 2 h. The

mixtures were rotated at 4°C for another 2 h after adding 10 μl nProtein A Sepharose pre-blocked with 1% BSA in PBS. The beads were washed 3 times with 0.15 M KCl Base Buffer (20 mM Tris, 150 mM KCl, 5 mM $MgCl_2$, 0.2 mM PMSF, 10% glycerol, 0.1% Tween-20, pH 8.0, supplemented with protease inhibitors).

For the Brd4 and MCV LT affinity analysis, MCV LT was Co-IPed with Brd4 antibody as described above. The Brd4-MCV LT complex isolated on nProtein A Sepharose was washed with buffer C (20 mM Tris, 0.2 mM PMSF, 0.1 mM EDTA, pH 8.0, supplemented with protease inhibitors and either 150, 200, 300, 500 or 1000 mM NaCl). Both dissociated MCV LT and the complex remained on the Sepharose were analyzed by Western blotting.

For anti-FLAG IP, cells harvested at 48 h p.t. were resuspended in the high salt buffer (10 mM HEPES, 500 mM NaCl, 3 mM $MgCl_2$, 0.2 mM PMSF and 1 mM DTT, pH 7.9, supplemented with protease inhibitors), passed through a 22-gauge needle ten times and extracted at 4°C for 1 h with rotation. After centrifugation at 14,000 rpm for 15 min, the supernatants were diluted in the low salt concentration buffer (10 mM HEPES, 10 mM NaCl, 3 mM $MgCl_2$, 0.2 mM PMSF and 1 mM DTT, pH 7.9, supplemented with protease inhibitors) to reach the physiological salt concentration. Samples were mixed with 10 μl of anti-FLAG M2 agarose beads (Sigma) pre-blocked with 1% BSA in PBS and rotated at 4°C for 4 h. The beads were washed three times with PBS containing 0.2 mM PMSF and protease inhibitors.

IPed proteins were eluted with 30 μl of sample buffer. Aliquots (10 μl) were resolved on a SDS-PAGE gel. Proteins were transferred to Immobilon-P (Millipore) and blotted with specific antibody to detect the protein of interest (ECL detection).

For GST pull down, GST and GST fusion proteins were produced in *E. coli* using the respective pGEX plasmids and immobilized on glutathione-agarose beads (Sigma). The beads were incubated with nuclear extracts prepared as described above or ^{35}S Met-labeled proteins produced by *in vitro* transcription/translation in reticulocyte lysate (Promega). After incubation at 4°C for 4h, the beads were washed 3 times with 0.15M KCl Base Buffer and eluted with 30 μl of SDS-PAGE sample buffer. An aliquot of each eluate (10 μl) was analyzed by autoradiography or Western blotting.

Supplemental References

1. Schowalter RM, Pastrana DV, Buck CB (2011) Glycosaminoglycans and sialylated glycans sequentially facilitate Merkel cell polyomavirus infectious entry. PLoS Pathog 7: e1002161.
2. Yan J, Li Q, Lievens S, Tavernier J, You J (2010) Abrogation of the Brd4-positive transcription elongation factor B complex by papillomavirus E2 protein contributes to viral oncogene repression. J Virol 84: 76-87.
3. Schowalter RM, Pastrana DV, Pumphrey KA, Moyer AL, Buck CB (2010) Merkel cell polyomavirus and two previously unknown polyomaviruses are chronically shed from human skin. Cell Host Microbe 7: 509-515.
4. You J, Srinivasan V, Denis GV, Harrington WJ, Jr., Ballestas ME, et al. (2006) Kaposi's sarcoma-associated herpesvirus latency-associated nuclear antigen interacts with bromodomain protein Brd4 on host mitotic chromosomes. J Virol 80: 8909-8919.
5. Kwun HJ, Guastafierro A, Shuda M, Meinke G, Bohm A, et al. (2009) The minimum replication origin of merkel cell polyomavirus has a unique large T-antigen loading architecture and requires small T-antigen expression for optimal replication. J Virol 83: 12118-12128.
6. Wu SY, Lee AY, Hou SY, Kemper JK, Erdjument-Bromage H, et al. (2006) Brd4 links chromatin targeting to HPV transcriptional silencing. Genes Dev 20: 2383-2396.
